# Thymic selection of the T cell receptor repertoire is biased toward autoimmunity in females

**DOI:** 10.7554/eLife.109041

**Published:** 2026-08-20

**Authors:** Hélène Vantomme, Valentin Quiniou, Leslie Adda, Charline Jouannet, Vanessa Mhanna, Celine Albalaa, Pierre Barennes, Nicolas Coatnoan, Vimala Diderot, Johanna Dubois, Gwladys Fourcade, Kenz Le Gouge, Otriv Frédéric Nguekap Tchoumba, Martin Pezous, Paul Stys, Adrien Six, Encarnita Mariotti-Ferrandiz, David Klatzmann

**Affiliations:** 1 Immunology, Immunopathology, Immunotherapy i3 Lab, INSERM UMRS-959, Sorbonne Université Paris France; 2 Biotherapy (CIC-BTi) and Inflammation-Immunolopathology-Biotherapy Department (i2B), Assistance Publique -Hôpitaux de Paris (AP-HP) Paris France; 3 Institut Universitaire de France Paris France; University of Toronto Canada; National Institute of Immunology India

**Keywords:** central tolerance, adaptive immunity, sex dimorphism, immune repertoire profiling, polyspecificity, Human

## Abstract

Women represent about 80% of patients with autoimmune diseases. This may partly result from sex-based differences in T cell receptor (TCR) selection during thymocyte development, potentially influenced by hormones and the lower expression of the Autoimmune Regulator (AIRE) transcription factor in females. To investigate this, we analyzed sex-specific differences in TCR generation and selection. We examined TCR repertoires in double-positive thymocytes and single-positive thymic cells, including CD8^+^ and CD4^+^ effector T cells and regulatory T cells (Tregs), derived from male and female organ donors. Minimal sex-based differences were observed in V and J gene usage, and there were no notable differences in TCR repertoire diversity, complementarity-determining region 3 (CDR3) length, amino acid composition, or network structure. No TCR sequences were exclusive to either sex. However, female effector T cells exhibited a significantly higher prevalence of TCRs specific to self-antigens implicated in autoimmunity compared to males, while female Tregs showed a reduced frequency of such TCRs. These differences were not observed for TCRs targeting self-antigens unrelated to autoimmunity or antigens associated with cancer or viruses. Our findings identify a sex-specific imbalance in thymic selection of TCRs with autoimmunity-associated specificities, providing mechanistic insight into the increased susceptibility of women to autoimmune diseases.

## Introduction

Several studies have highlighted a sex imbalance in diseases involving the immune system. This is particularly evident in the case of autoimmune diseases (AIDs), where 80% of affected individuals are women, and the severity of the condition varies between the sexes (Libert et al., 2010; Golden and Voskuhl, 2017; Sokka et al., 2009). In addition, men are more severely affected by infectious diseases such as COVID-19 and tuberculosis (Li et al., 2020; Hertz and Schneider, 2019). The response to preventive immunotherapies such as vaccination, and to curative treatments, like immune checkpoint inhibitors or anti-TNF alpha, is also sex dependent (Klein and Morgan, 2020). These observations indicate the presence of intrinsic biological differences in immune responses between males and females.

A variety of factors have been identified that could help explain the observed sex-based differences. Among these, sex hormones have been shown to influence immune responses by acting on immune cells that express specific receptors to these hormones (Lee and Chang, 2003; Chen et al., 2022; Scarpin et al., 2009). Sex hormones could directly influence the adaptive immune response at the level of thymocyte differentiation and selection by acting on the expression of Autoimmune Regulator (AIRE) in thymic epithelial cells. AIRE is responsible for the thymic expression of otherwise tissue-specific antigens, contributing to both the negative selection of effector cells and the positive selection of Tregs that recognize such antigens (Klein et al., 2014). Research has shown that testosterone increases AIRE expression in medullary thymic epithelial cells, whereas estradiol decreases it (Zhu et al., 2016; Dragin et al., 2016). Furthermore, a recent study has shown that the transcriptomic profiles of these specialized antigen-presenting cells in the thymus differ between males and females (Stankiewicz et al., 2025). This suggests that there may be differences in the selection of the T cell receptor (TCR) repertoire between males and females.

Despite these findings, there is still limited knowledge about potential differences in the TCR repertoire between the sexes. The generation of the TCR is a key process in T-cell development that takes place in the thymus. The TCR consists of two chains, alpha (TRA) and beta (TRB), each of which results from a random genetic rearrangement involving the V (variable), D (diversity) [for the TRB], and J (joining) genes. The combination of these gene segments is accompanied by insertions and deletions, creating a highly diverse region, the Complementarity Determining Region 3 (CDR3; Murugan et al., 2012). It is this region of the TCR that predominantly interacts with the peptide, a critical interaction for antigen recognition and subsequent T cell activation. The ability to specifically recognize diverse antigens via the CDR3 is central to the efficiency and specificity of the adaptive immune response.

TCRs are randomly generated, which means that while some may be useful, others may be harmful by recognizing self-antigens and causing autoimmunity. The current understanding is that during thymocyte development, selection is based on the avidity of their TCRs for antigens presented by cortical and medullary thymic epithelial cells. Thymocytes expressing TCRs with insufficient avidity fail to receive survival signals and undergo death by neglect during positive selection, whereas those with excessive avidity are actively eliminated via negative selection to ensure self-tolerance. In contrast, thymocytes with intermediate avidity are positively selected and mature into single-positive (SP) cells, either CD4 +or CD8+. Thymocytes with the highest of these intermediate affinities develop in Tregs. Given AIRE’s pivotal function in these processes, sex differences in AIRE expression may significantly influence thymic selection.

The TCR repertoire of female and male peripheral T cells has shown that their diversity, particularly that of the TRB repertoire, is influenced not only by the age of the individuals but also by their sex (Yoshida et al., 2017; Trofimov et al., 2022; Mika et al., 2023; Gong et al., 2021). However, another study showed no association between the sex of individuals and the diversity of their TCR repertoire (Qi et al., 2014). Nevertheless, the study of peripheral blood TCRs reflects that of an immune system that has undergone multiple immune responses. For example, observed differences between males and females in their responses to self-antigens associated with autoimmunity could indicate either the cause (the TCRs drive the disease) or the consequence (the TCRs are expanded because of the disease).

To assess whether there are significant differences in the generation and thymic selection of the TCR repertoire between males and females, we therefore focused on analyzing the TCR repertoires of thymocytes. These cells represent the latest stage in T cell development and have not yet been involved in immune responses. Their repertoires thus represent the mechanisms of TCR generation for the analysis of CD4/CD8 double-positive (DP) cells and of TCR selection for CD4 and CD8 SP T cells. We generated a unique data set comprising the sequences of the TRA and TRB chains of DP cells, CD8 and CD4 SP effector cells, and CD4 SP Tregs from organ donors, both infants and adults. The analysis of TRA and TRB repertoires using state-of-the-art strategies revealed no sex differences in repertoire generation (DP cell repertoire), but an enrichment in females for CD8 SP T cells bearing CDR3 associated with specificities related to autoimmunity.

## Results

**Figure 1.**
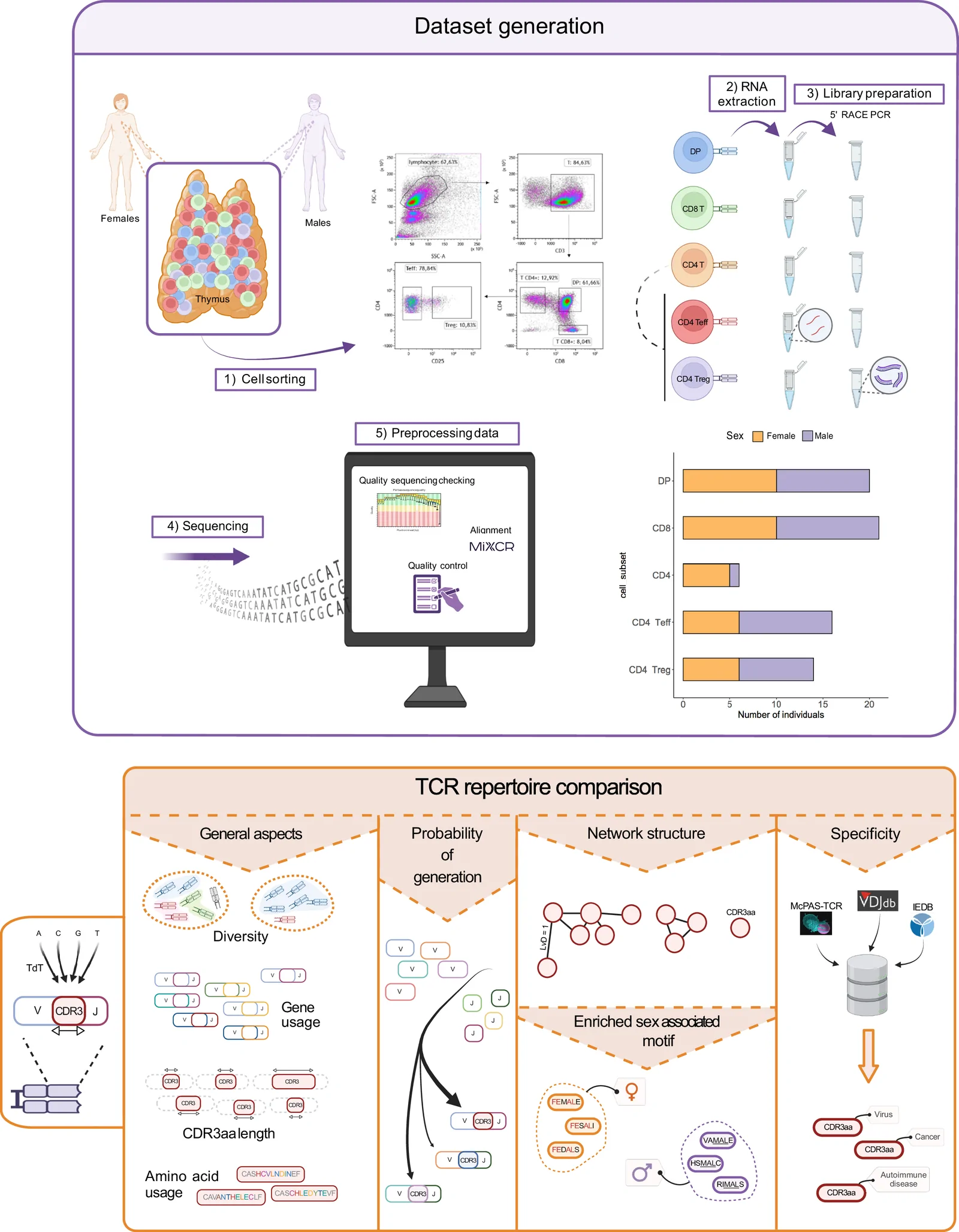
Schematic overview of the generation of the thymic T cell receptor (TCR) dataset and the analytical pipeline. Top panel: Generation of the thymic TCR dataset. From deceased human thymuses of males and females, we isolated key T-cell subtypes through cell sorting. These subtypes included double-positive (DP) cells (CD3+CD4+CD8+), single-positive (SP) CD8 + cells (CD3+CD4 CD8+), SP CD4 + cells (CD3+CD4+CD8-) that were further separated into T effector (Teff, CD3+CD4+CD8-CD25-) and Treg (CD3+CD4+CD8 CD25+) cells. TCR libraries were generated from the RNA of each cell population using rapid amplification of cDNA ends by PCR (5'RACE PCR). Following sequencing, data preprocessing involved quality sequencing checks, contig alignment, and quality control. The final dataset comprised DP samples (n=20; male-to-female ratio of 1:1), SP CD8+samples (n=21; 1.1:1), SP CD4+samples (n=6; 1:5), SP CD4 Teff samples (n=16; 1.67:1), and SP CD4 Treg samples (n=14; 1.33:1). Males are depicted in violet and females in orange. Bottom panel: Analytical pipeline. We compared the TCR repertoires of males and females across various dimensions. We evaluated general aspects of the TCR repertoire were evaluated, including diversity, gene usage, CDR3aa length distribution, and aa usage within the CDR3 region. Additionally, we analyzed the probability of sequence generation and the TCR repertoire structure based on CDR3aa sequence similarity. We identified differentially expressed TRB CDR3aa motifs between sexes and analyzed TRB CDR3aa sequence specificity. Figure 1 was created with BioRender.com.

We analyzed thymic TCRαβ repertoires from 22 human organ donors (11 females, 11 males, aged between 73 days and 64 years; Figure 1, Figure 1—figure supplement 1). For each donor, CD3 +CD4+CD8+ DP, CD3 +CD4 CD8+CD8 SP, and CD3 +CD4+CD8 CD4 SP thymocytes were sorted by flow cytometry. In 16 donors, CD4 SP cells were further subdivided into CD25- conventional CD4 Teff and CD25 +CD4 Treg subsets, whereas in the remaining six donors, total CD4 SP cells were analyzed as Teff-enriched populations. This resulted in 20 DP, 21 CD8 SP, 22 CD4 Teff SP, and 14 CD4 Treg SP samples (Figure 1, Figure 1—figure supplement 1). Bulk TCR sequencing was then performed on each cell subset, with the aim of characterizing the TRA and TRB repertoires. Rank–frequency plots of clonotype abundances displayed comparable heavy-tailed distributions across donors, without any obvious systematic differences between males and females (Figure 1—figure supplement 2).

**Figure 1—figure supplement 1.**
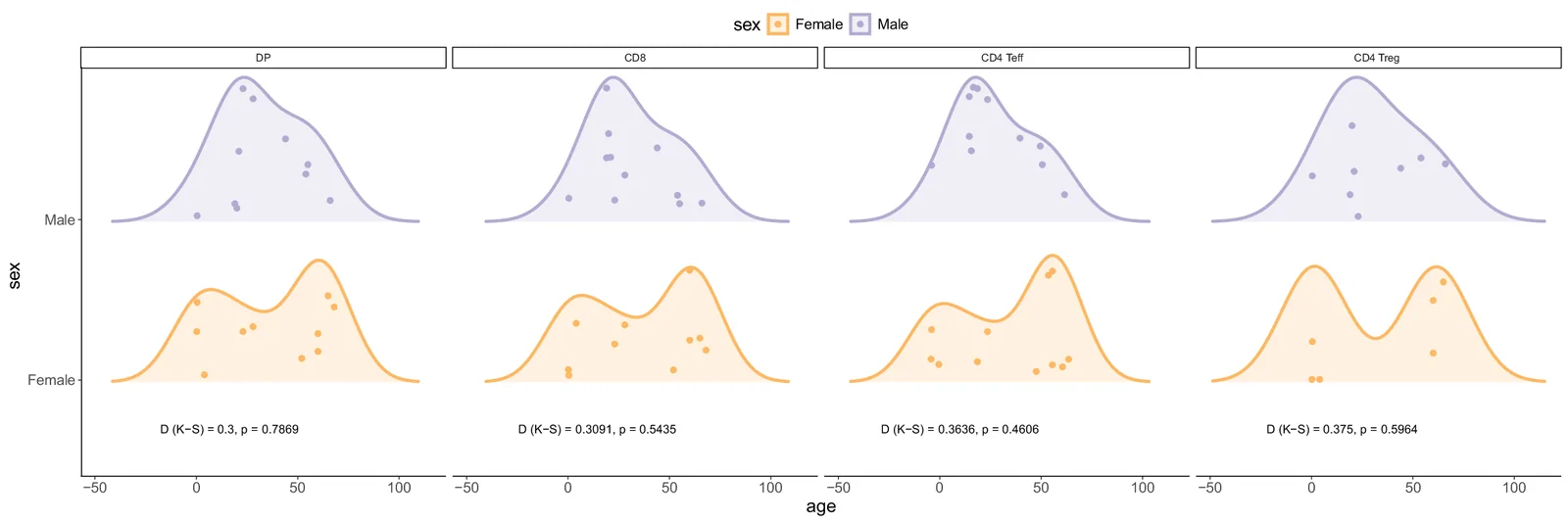
Age distribution of male and female donors is comparable across thymic subsets. Age of individual donors plotted separately for females (orange) and males (violet) for each thymic cell subset: double-positive (DP) thymocytes (n=20), CD8 single-positive (CD8) (n=21), CD4 effector single-positive (CD4 Teff) (n=22), and CD4 regulatory T-cell single-positive (CD4 Treg) (n=14). For each subset, a two-sample Kolmogorov–Smirnov (K–S) test was performed; the D statistic and corresponding p-value are displayed below each panel. In all cases, no significant difference was detected, indicating balanced age distributions between sexes for every subset.

**Figure 1—figure supplement 2.**
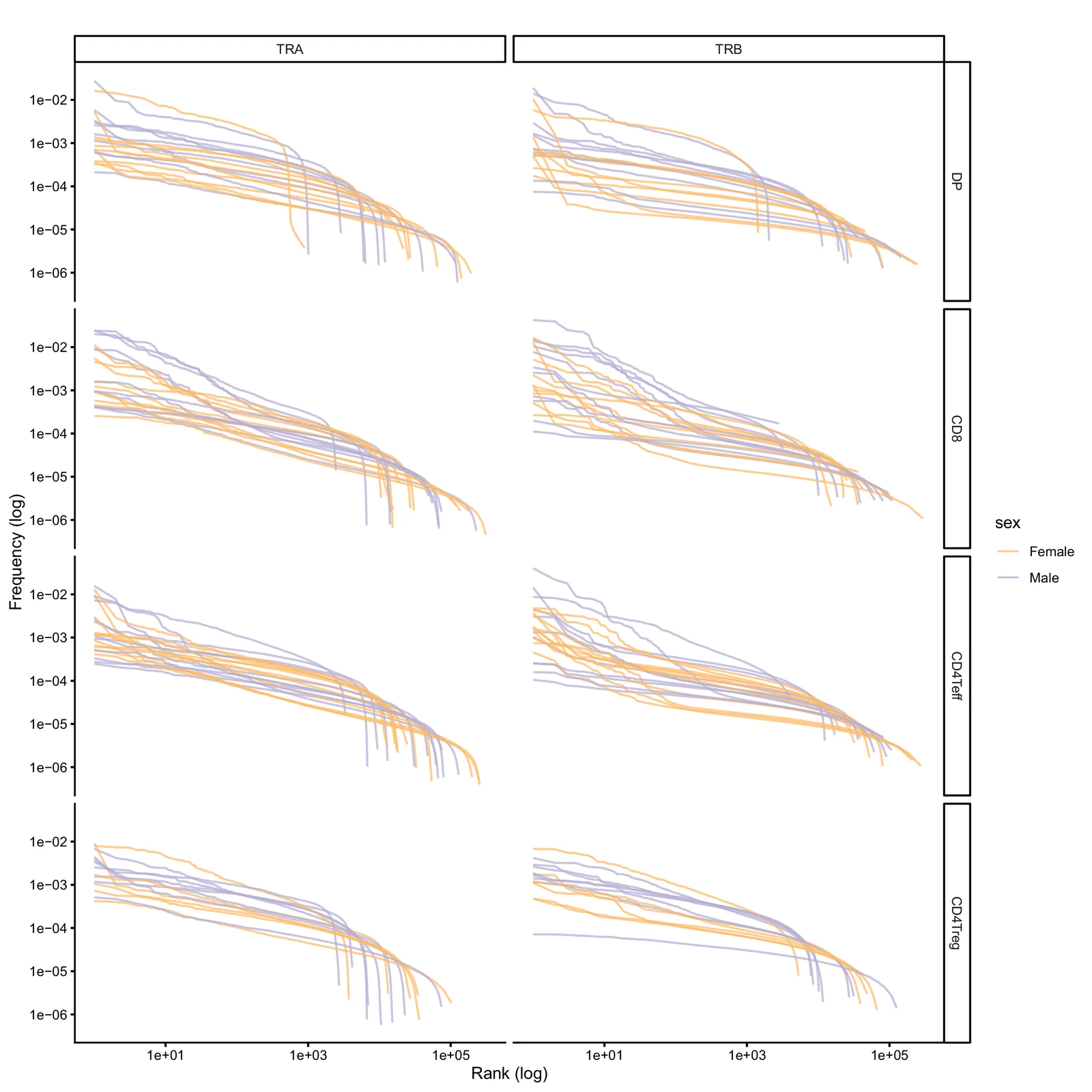
Rank–frequency distributions of thymic TRA and TRB clonotypes. Log–log rank–frequency plots of unique TRA (left) and TRB (right) clonotypes are shown for each donor and thymic subset (double positive [DP] – n=20, CD8 – n=21, CD4 Teff – n=22, CD4 Treg – n=14, from top to bottom). For each sample, clonotypes are ranked by decreasing abundance (x-axis, log scale), and their corresponding relative frequencies are plotted (y-axis, log scale). Curves display broadly comparable shapes across donors, with no obvious systematic differences between males and females, indicating similar clonotype abundance distributions and sampling depth across sexes.

### Minimal sex-based differences in TCR V and J gene usage across thymic cell subsets

The first level of diversity in generating TCRs is the usage of V and J genes (Davis and Bjorkman, 1988). We analyzed the usage of TRA and TRB V and J genes and their combinations in each thymocyte population (Figure 1).

**Figure 2.**
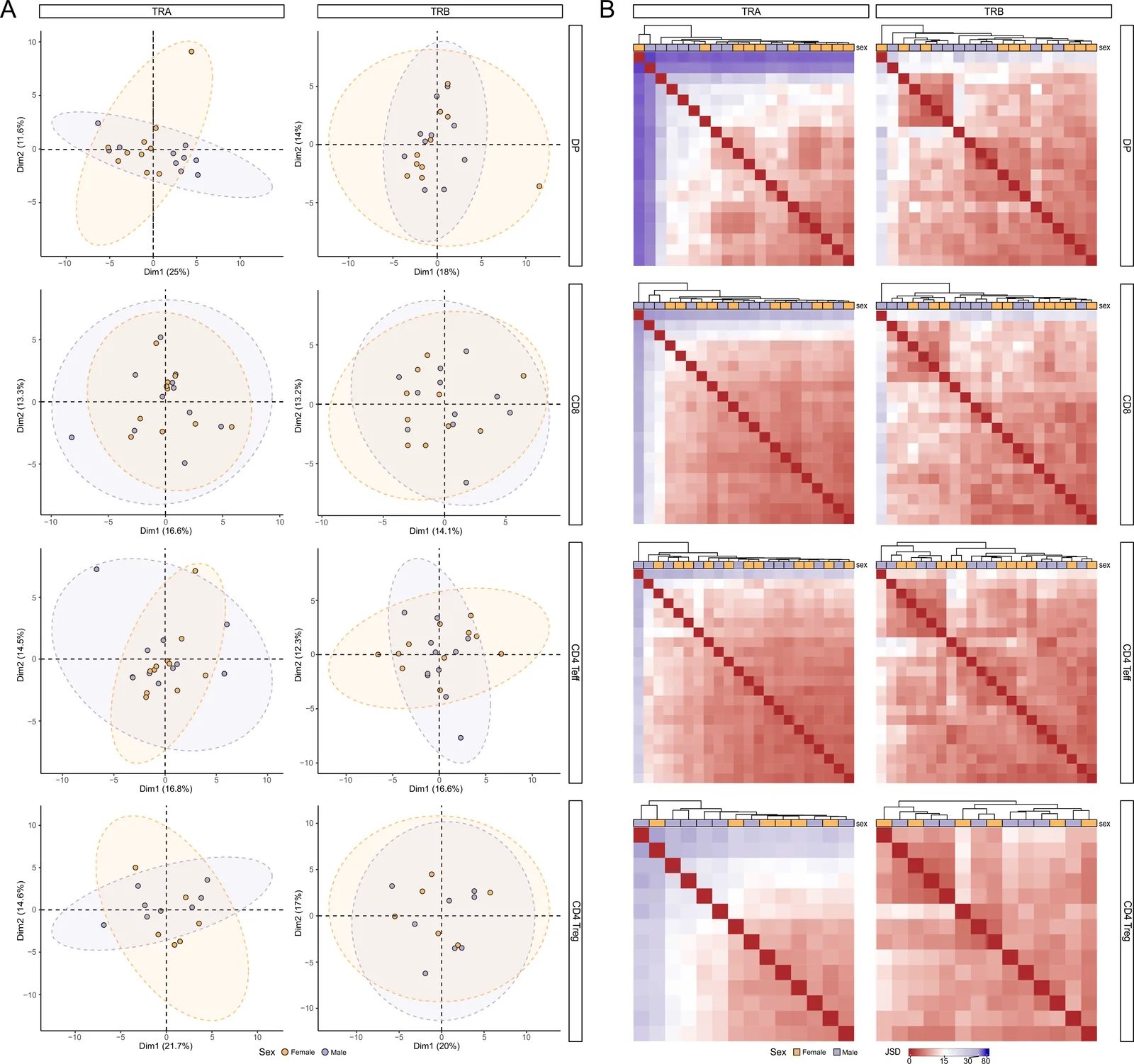
Comparable overall T cell receptor (TCR) gene usage between males and females. (**A**) Principal component analysis (PCA) derived from the distribution of TRAV (left) and TRBV (right) gene usage frequencies across sex groups (males vs females), showing results for double-positive (DP; n=20), CD8 (n=21), CD4 Teff (n=22), and CD4 Treg (n=14) cells (displayed from top to bottom). Each point on the graph represents an individual. Ellipses indicate 95% confidence intervals. (**B**) Heatmap showing the Jensen-Shannon Divergence (JSD) score between samples, derived from the distributional usage of TRAV-TRAJ (left) and TRBV-TRBJ (right) gene associations in DP (n=20), CD8 (n=21), CD4 Teff (n=22), and CD4 Treg (n=14) cells (displayed from top to bottom). Hierarchical clustering was performed using the Euclidean distance and the complete linkage method. Males are shown in violet and females in orange.

Males and females had a relatively similar V gene usage, as evidenced by the lack of a clear separation in the Principal Component Analysis (PCA), for both TRA and TRB, and across all cell subtypes studied (Figure 2A). However, we observed some individual differences in V and J gene usage between males and females, with some being specific to certain cell subtypes (Figure 2—figure supplements 1–4), and others observed across multiple cell subtypes (Figure 2—figure supplements 1–4). For example, TRBV6-5 was found to be more highly expressed in females compared to males in DP cells (Figure 2—figure supplement 1C) and CD8 SP cells (Figure 2—figure supplement 2C).

**Figure 2—figure supplement 1.**
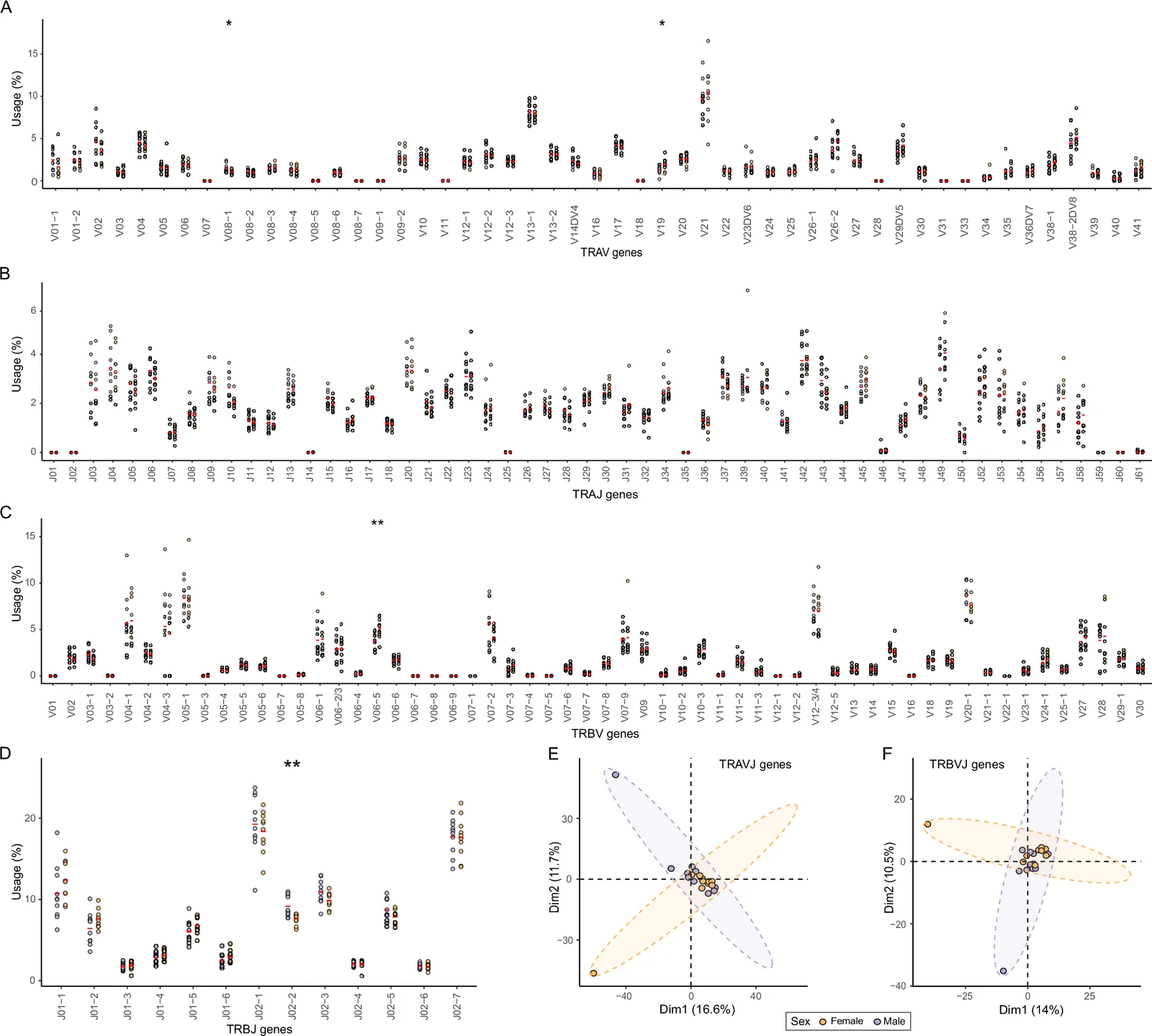
Some differential V and J gene usage in T cell receptor (TCR) repertoires of double-positive (DP) cells between males and females. (**A–D**) Frequencies of TRAV (**A**), TRAJ (**B**), TRBV (**C**), and TRBJ (**D**) gene usage between males and females (n = 10 per group). Each dot represents one donor. Horizontal red lines indicate the mean for each sex group. Statistical comparisons between groups were performed using two-sided Wilcoxon test. Asterisks indicate significant differences between males and females based on the test p-value (*: p<0.05, **: p<0.01). (**E–F**) Principal component analysis (PCA) derived from the distribution of the frequency of usage of TRAV-TRAJ (**E**) and TRBV-TRBJ (**F**) gene associations across donors. Each point represents an individual. Ellipses show 95% confidence intervals. Males are depicted in violet (n=10) and females in orange (n=10).

**Figure 2—figure supplement 2.**
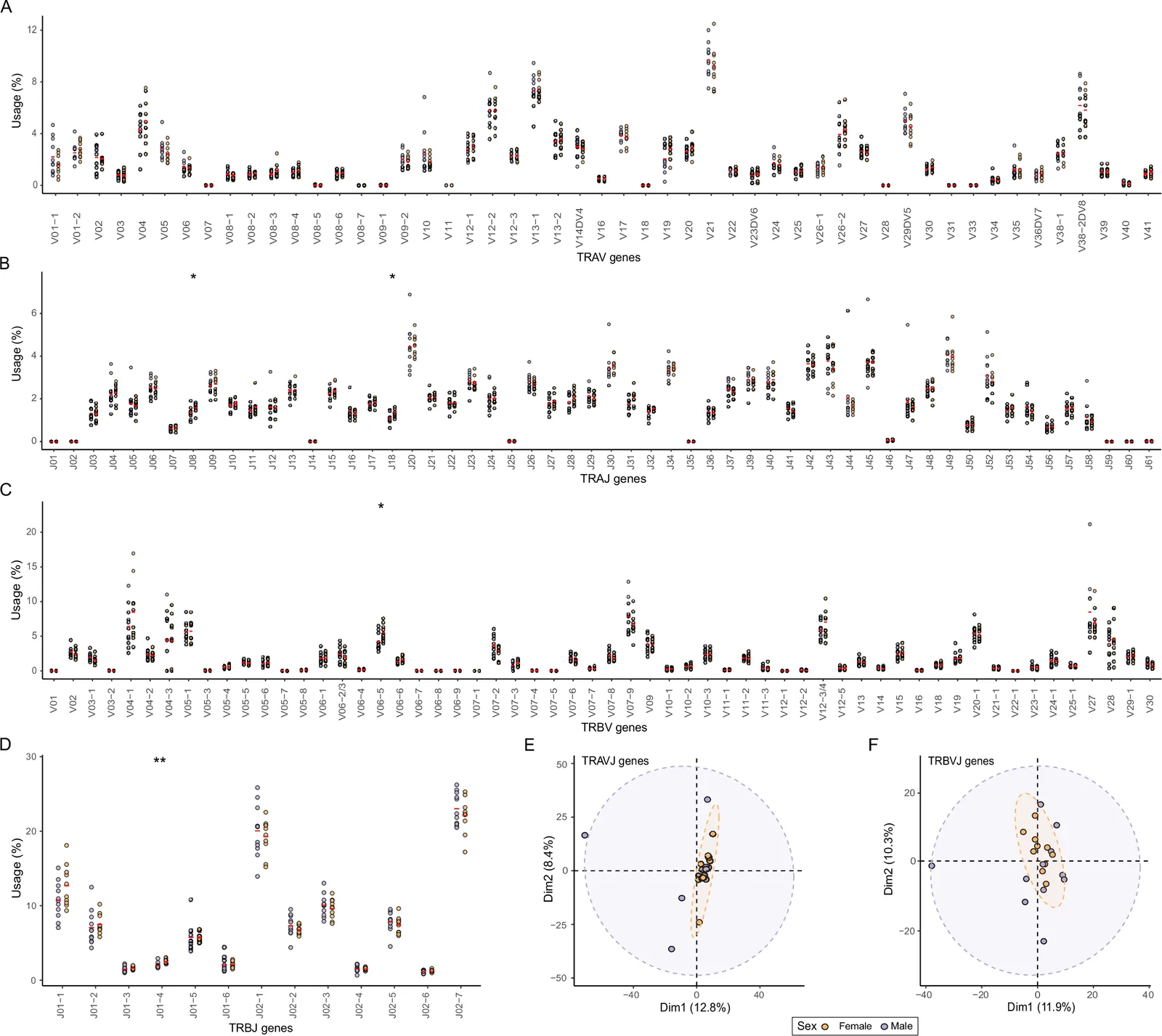
Some differential V and J gene usage in T cell receptor (TCR) repertoires of CD8 cells between males and females. (**A–D**) Frequencies of TRAV (**A**), TRAJ (**B**), TRBV (**C**), and TRBJ (**D**) gene usage between males (n = 11) and females (n = 10). Each dot represents one donor. Horizontal red lines indicate the mean for each sex group. Statistical comparisons between groups were performed using two-sided Wilcoxon test. Asterisks indicate significant differences between males and females based on the test p-value (*: p<0.05, **: p<0.01). (**E–F**) Principal component analysis (PCA) derived from the distribution of the frequency of usage of TRAV-TRAJ (**E**) and TRBV-TRBJ (**F**) gene associations across donors. Each point represents an individual. Ellipses show 95% confidence intervals. Males are depicted in violet (n=11) and females in orange (n=10).

**Figure 2—figure supplement 3.**
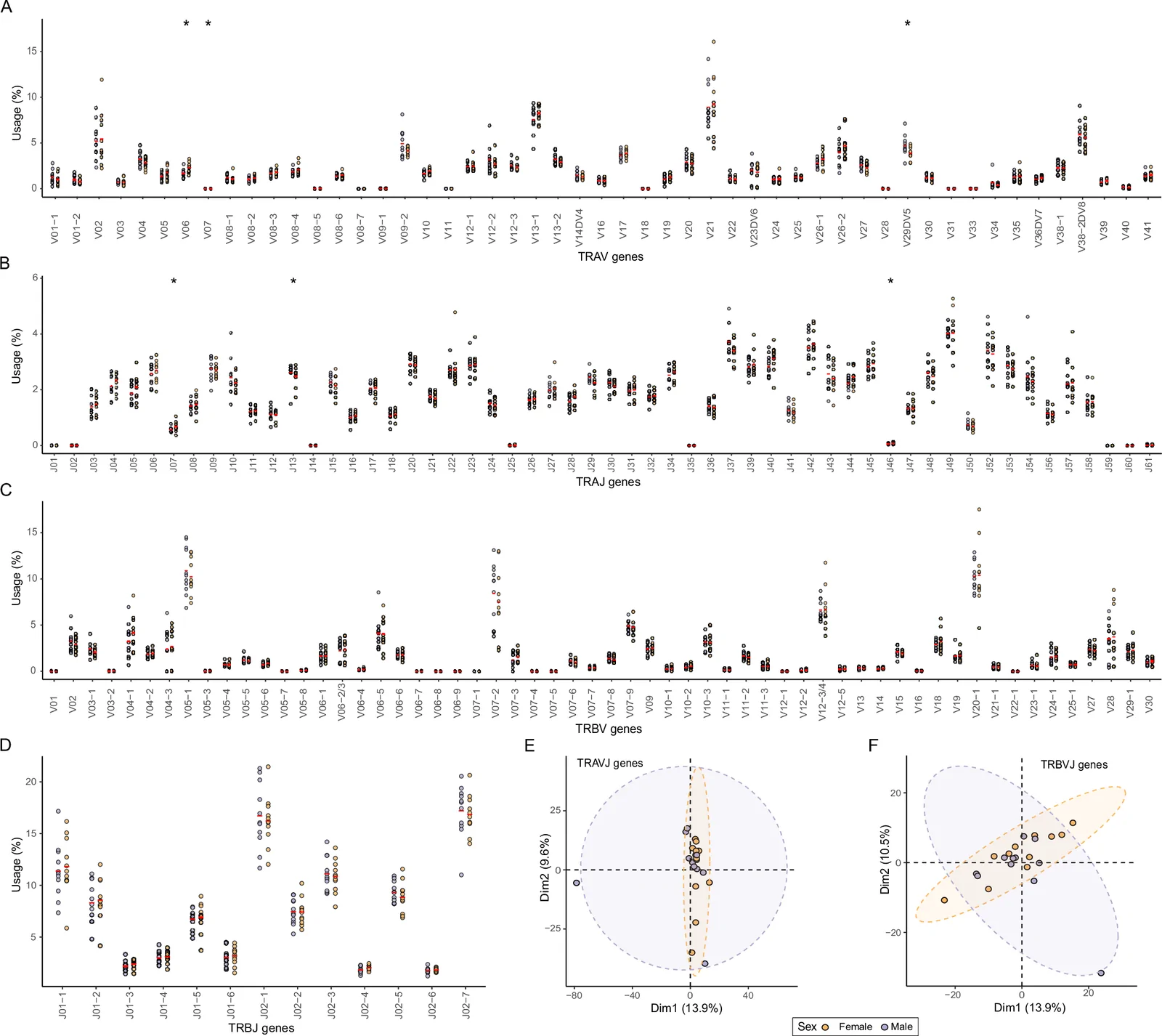
Some differential V and J gene usage in T cell receptor (TCR) repertoires of CD4 Teff cells between males and females. (**A–D**) Frequencies of TRAV (**A**), TRAJ (**B**), TRBV (**C**), and TRBJ (**D**) gene usage between males and females (n = 11 per group). Each dot represents one donor. Horizontal red lines indicate the mean for each sex group. Statistical comparisons between groups were performed using two-sided Wilcoxon test. Asterisks indicate significant differences between males and females based on the test p-value (*: p<0.05, **: p<0.01). (**E–F**) Principal component analysis (PCA) derived from the distribution of the frequency of usage of TRAV-TRAJ (**E**) and TRBV-TRBJ (**F**) gene associations across donors. Each point represents an individual. Ellipses show 95% confidence intervals. Males are depicted in violet (n=11) and females in orange (n=11).

**Figure 2—figure supplement 4.**
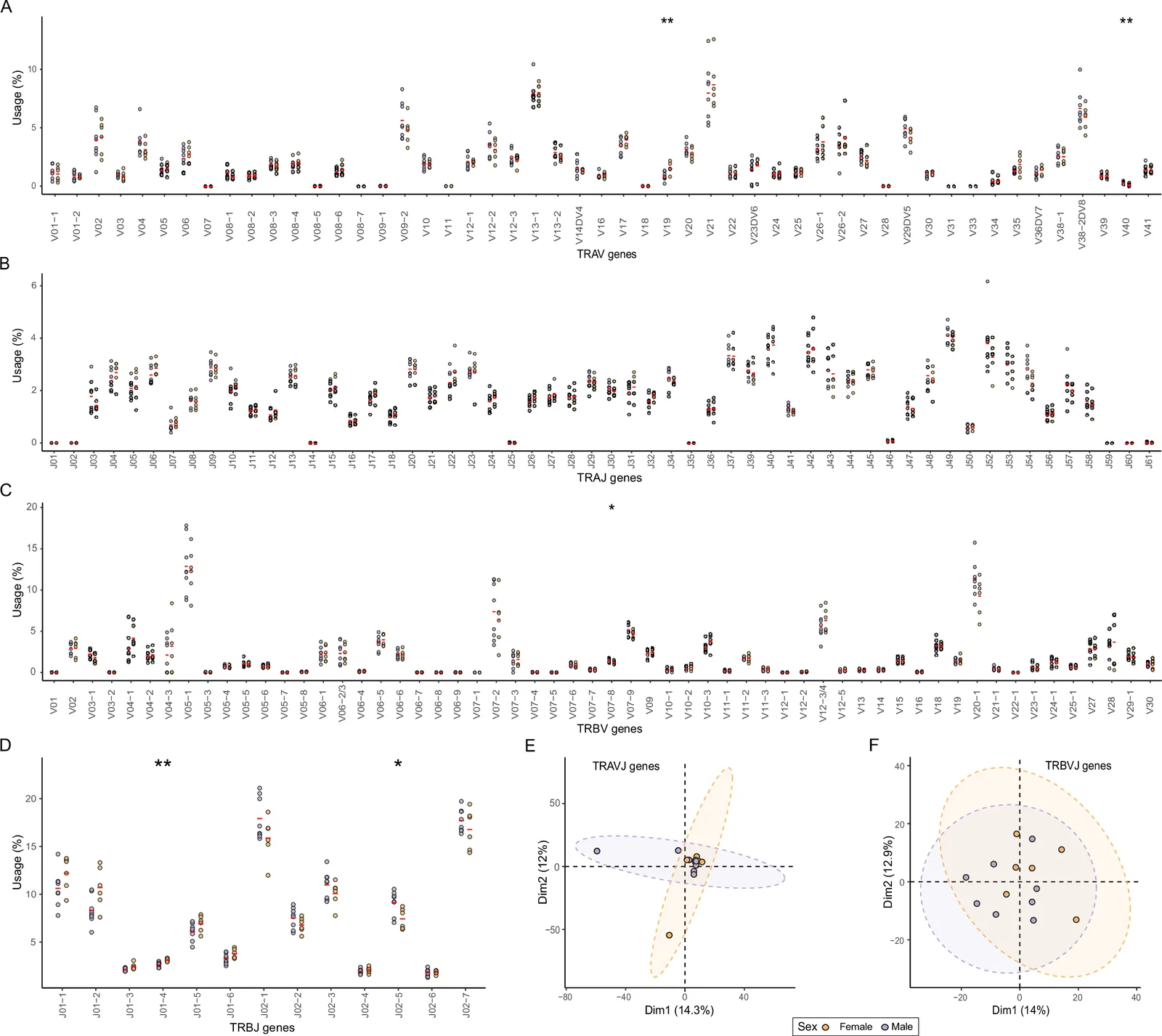
Some differential V and J gene usage in T cell receptor (TCR) repertoires of CD4 Treg cells between males and females. (**A–D**) Frequencies of TRAV (**A**), TRAJ (**B**), TRBV (**C**), and TRBJ (**D**) gene usage between males (n = 8) and females (n = 6). Each dot represents one donor. Horizontal red lines indicate the mean for each sex group. Statistical comparisons between groups were performed using two-sided Wilcoxon test. Asterisks indicate significant differences between males and females based on the test p-value (*: p<0.05, **: p<0.01). (**E–F**) Principal component analysis (PCA) derived from the distribution of the frequency of usage of TRAV-TRAJ (**E**) and TRBV-TRBJ (**F**) gene associations across donors. Each point represents an individual. Ellipses show 95% confidence intervals. Males are depicted in violet (n=8) and females in orange (n=6).

In the following step, we compared the usage of TRAV-TRAJ and TRBV-TRBJ gene combinations across all individuals using the Jensen-Shannon Divergence (JSD) score (Figure 2B). The hierarchical clustering showed no clear separation between male and female individuals for any of the chain or cell subtype, indicating no significant differences in the gene combination usage between males and females (Figure 2B). A similar observation was made when directly analyzing the frequency of these VJ gene combinations (Figure 2—figure supplements 1E, F–4E, F).

### Comparable TCR repertoire diversity between males and females

A high diversity of the TCR repertoire is essential for generating a broad potential reactivity against a variety of antigens. We thus compared the TCR repertoire diversity between males and females. To achieve this objective, we analyzed Rényi diversity curves (from zero to infinite α parameters) that measure various aspects of TCR repertoire diversity. At different α values, the Rényi index places more or less weight on the contribution of the most frequent clonotypes. Higher α values highlight the most prevalent clonotypes, thereby revealing the effect of expanded clones on overall diversity.

**Figure 3.**
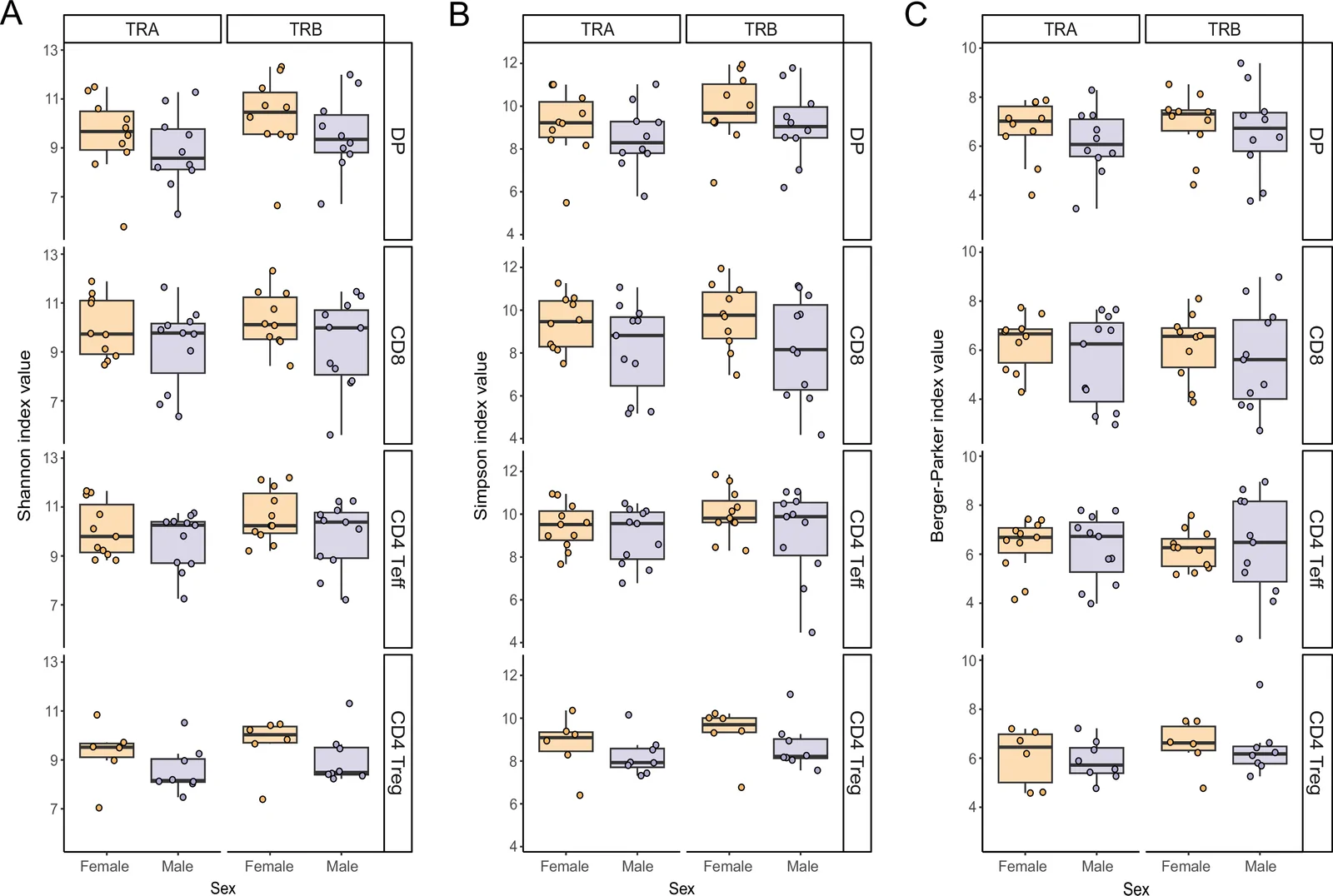
Comparable thymic T cell receptor (TCR) repertoire diversity between males and females. Boxplots display Shannon (**A**), Simpson (**B**), and Berger-Parker (**C**) index values for TRA (left) and TRB (right), across thymic T cell subtypes, displayed from top to bottom, in double-positive (DP; n=20), CD8 (n=21), CD4 Teff (n=22), and CD4 Treg (n=14). Each point on the graph represents the median value from 50 rarefactions per sample. Statistical analysis (two-sided Wilcoxon test) showed no significant sex bias in TCR repertoire diversity (p>0.05). Males are shown in violet and females in orange.

Rényi curves showed comparable diversities between sexes in DP and CD4 Teff cells, for both TRA and TRB, and a difference in CD8 and CD4 Treg cells (Figure 3—figure supplement 1). In CD8 cells, the curves begin to diverge at low α values (Figure 3—figure supplement 1), with an earlier inflection in males, indicating a less balanced distribution of TCR clones. This suggests that in males, fewer clones dominate the repertoire at lower diversity thresholds compared to females. In Tregs, while the inflection point is similar, the difference seems to be characterized by higher diversity and richness in females compared to males (Figure 3—figure supplement 1). However, no significant differences were observed for the Shannon diversity index (α=1), the Simpson index (α=2), and the Berger-Parker index (α=infinite), for both chains and all cell subtypes (Figure 3). These results indicate that the diversity of thymic TCR repertoire is similar between males and females.

**Figure 3—figure supplement 1.**
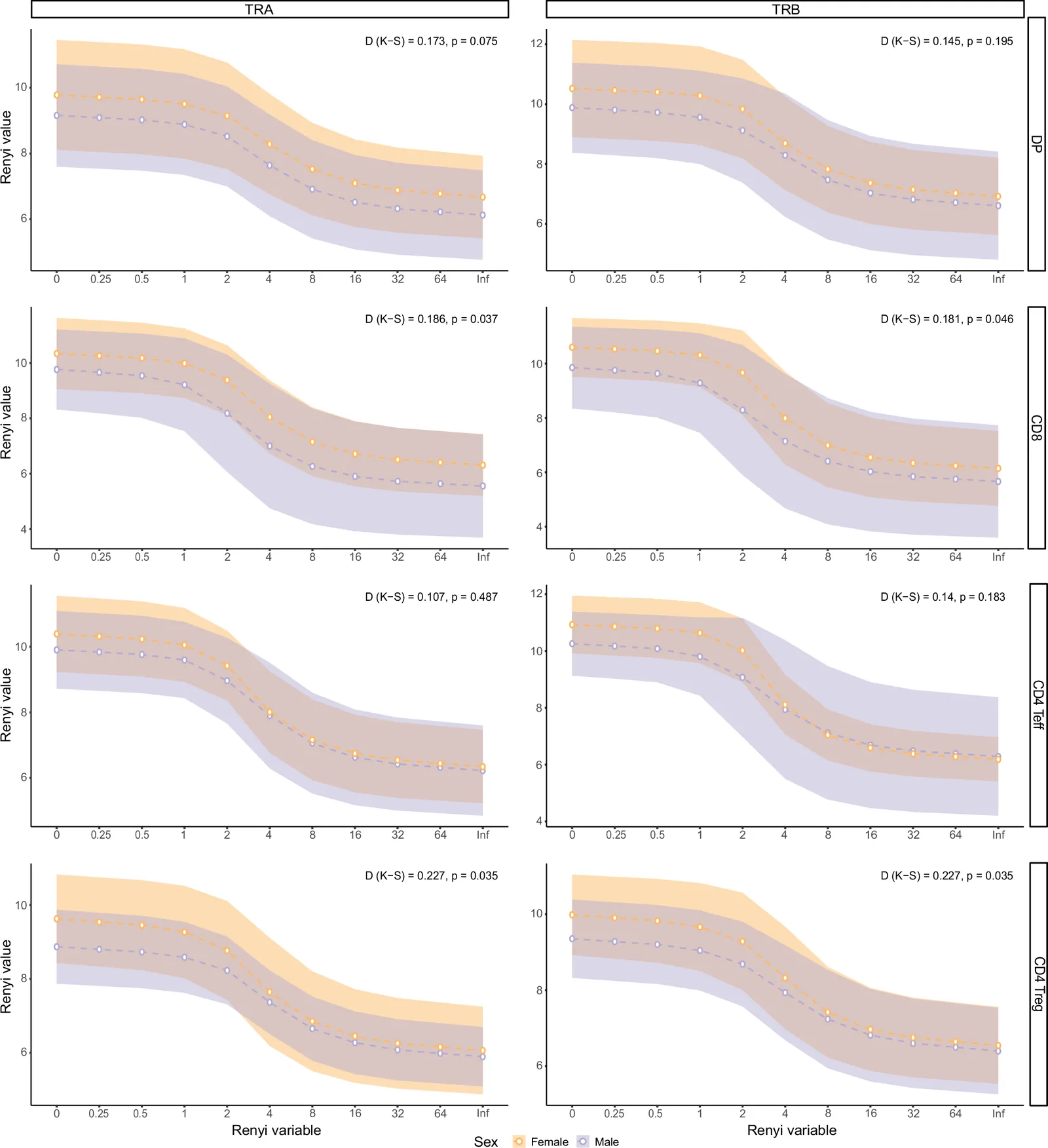
Minimal differences in diversity profile of CD8 and CD4 Treg thymic T cell receptor (TCR) repertoire between males and females. Diversity profile with Rényi diversity index values (α ranging from 0 to infinity) for all cell subsets (double-positive [DP] – n=20, SP CD8 – n=21, SP CD4 Teff – n=22, and SP CD4 Treg – n=14) and for both TRA (left) and TRB (right). Clonotypes of each sample were rarefied fifty times to their effective diversity number [i.e. $e^{Shannonindex}$] and the median of their Rényi values was used for analysis. Dotted lines and points show the mean value for each sex group, while the shaded area represents the SD for each sex group. The overall shape of the curves was compared statistically using the Kolmogorov-Smirnov test, with the D value and associated p-value indicated.

### Comparable CDR3aa length distribution in male and female TCR repertoires

Most of the repertoire diversity is generated by the random addition of nucleotides within the VDJ junctions, resulting in the hypervariable CDR3 region that interacts with the peptide-MHC complex and is mostly responsible for the TCR specificity. The CDR3 length has been shown to directly influence the TCR’s ability to interact with the peptide (Wang et al., 1998). We thus studied the distribution of CDR3 aa (CDR3aa) sequence lengths between males and females to identify potential TCR generation and/or selection biases (Figure 1).

**Figure 4.**
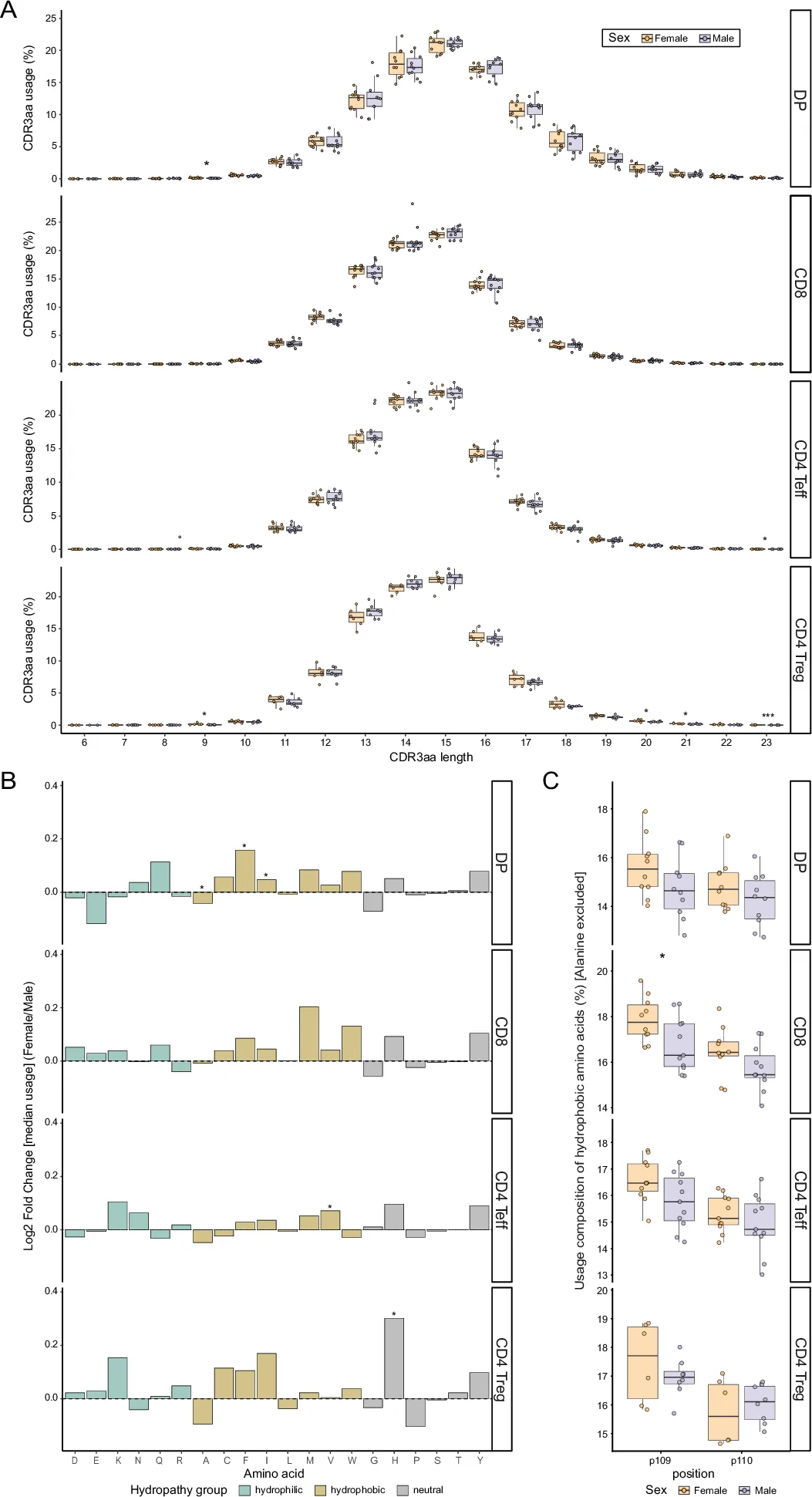
CDR3aa length and amino acid composition of TRB CDR3s in males and females. (**A**) Distribution of TRB CDR3 amino acid (CDR3aa) length usage in double-positive (DP; n=20), CD8 (n=21), CD4 Teff (n=22), and Treg (n=14) single-positive (SP) thymic cells, displayed from top to bottom. Asterisks indicate significant differences between males and females based on the two-sided Wilcoxon test p-value (*: p<0.05, **: p<0.01). (**B**) The data on amino acid (aa) usage between males and females are presented as the log2 fold change of the median per-donor usage in females over males for each aa in the p108 to p114 CDR3aa region for TRB. A line at log2 fold change = 0 is indicative of the direction of the difference in usage frequency. The bars are color-coded according to the hydropathy class of the aa as defined by the Kyte-Doolittle-based IMGT classification: neutral aa by gray, hydrophilic aa by blue-green, and hydrophobic aa by gold. Asterisks indicate statistical differences of usage between males and females based on the two-sided Wilcoxon test p-value (*: p<0.05, **: p<0.01). (**C**) Position-specific usage of hydrophobic aa (excluding alanine, due to its weak hydrophobicity) at IMGT positions p109 and p110 in TRB across thymic T cell subtypes, including DP (n=20), CD8 (n=21), CD4 Teff (n=22), and CD4 Treg (n=14). For each donor, the values represent the proportion of unique TRB CDR3aa sequences carrying a hydrophobic amino acid at the indicated position. Asterisks indicate significant sex differences based on the two-sided Wilcoxon test p-value (*: p<0.05), with a significant increase in hydrophobic usage at p109 in female CD8 SP cells. Males are depicted in violet and females in orange.

Our results showed that males and females have a comparable distribution of CDR3aa lengths for both TRA (Figure 4—figure supplement 1) and TRB (Figure 4A). CDR3aa sequences in TRA were shorter than those in TRB, as previously described in peripheral blood T cells (Yu et al., 2019; Carter et al., 2019). Although there were subtle differences in certain CDR3aa lengths between males and females, these variations were minimal, involving sequences that represent less than 2% of the total TCR repertoire (Figure 4A, Figure 4—figure supplement 1). Altogether, the overall distribution of CDR3aa length is comparable between males and females.

**Figure 4—figure supplement 1.**
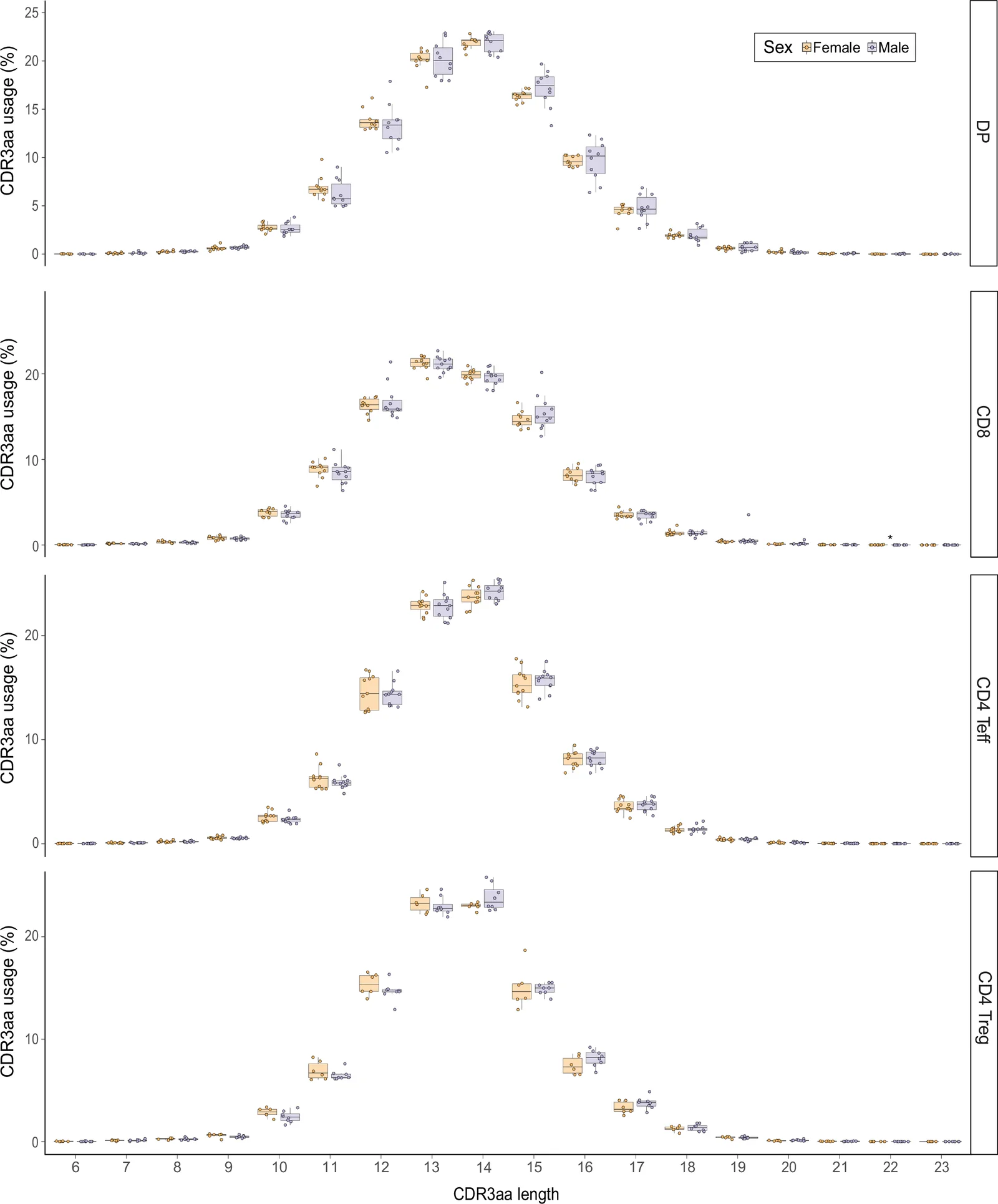
Comparable TRA CDR3aa length distribution between males and females. Distribution of CDR3aa length usage for TRA in all thymic T cell subsets, including double-positive (DP; n=20), CD8 (n=21), CD4 Teff (n=22), and CD4 Treg (n=14). For each donor, the values represent the proportion of unique TRB CDR3aa sequences carrying a hydrophobic amino acid at the indicated position. Asterisks indicate significant differences between males and females based on the two-sided Wilcoxon test p-value (*: p<0.05, **: p<0.01). Males are depicted in violet and females in orange.

**Figure 4—figure supplement 2.**
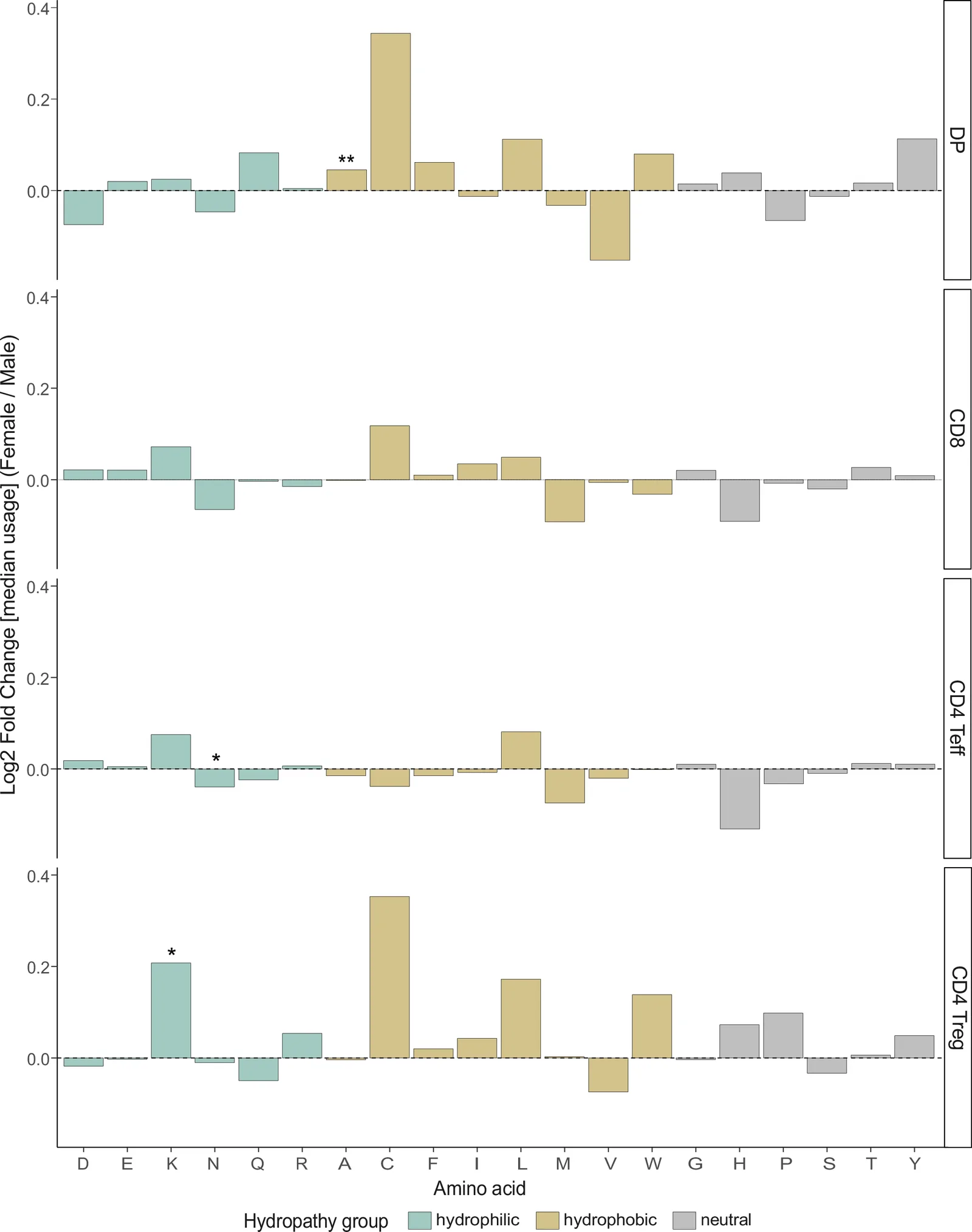
Some differences in TRA CDR3aa usage between males and females. The data on amino acid (aa) usage between males and females are presented as the log2 fold change of the median per-donor usage in females over males for each aa in the p108 to p114 CDR3aa region for TRA, across thymic T cell subsets: double-positive (DP; n=20), CD8 single-positive (SP; n=21), CD4 Teff SP (n=22), and CD4 Treg SP (n=14). A line at log2 fold change = 0 is indicative of the direction of the difference in usage frequency. The bars are color-coded according to the hydropathy class of the aa as defined by the Kyte-Doolittle-based IMGT classification: neutral aa by gray, hydrophilic aa by blue-green, and hydrophobic aa by gold. Asterisks indicate statistical differences of usage between males and females based on the two-sided Wilcoxon test p-value (*: p<0.05, **: p<0.01).

### Subtle differences in the composition of CDR3aa in the thymic TCR repertoires of males and females

We analyzed the aa usage within the FG loop of the CDR3, which is in contact with the peptide-MHC complex (Garcia et al., 1998). The classification of aa was determined based on their hydropathy properties, categorized as follows: hydrophilic, hydrophobic, or neutral (Pommié et al., 2004). We observed significant differences in the usage of some aa between males and females for both TRA (Figure 4—figure supplement 2) and TRB (Figure 4B). In DP cells, these differences predominantly involved hydrophobic aa: females exhibited higher alanine (A) usage in TRA and lower in TRB compared with males (Figure 4B, Figure 4—figure supplement 2). Additionally, females showed increased usage of phenylalanine (F) and isoleucine (I) in TRB (Figure 4B). Furthermore, other aa with varying hydropathy properties were differentially used between males and females in CD4 Teff and Treg populations (Figure 4B, Figure 4—figure supplement 2).

We then examined the usage of aa specifically at positions p109 and p110 of the TRB CDR3aa region, as hydrophobic aa at these two positions have been associated with a stronger recognition of self-antigens (Khosravi-Maharlooei et al., 2019; Lu et al., 2019; Stadinski et al., 2016). For each individual, we calculated the proportion of unique TRB CDR3aa carrying a hydrophobic aa (excluding alanine, which is only weakly hydrophobic) at these two positions. This positional analysis revealed a significant increase in the usage of hydrophobic amino acids at position p109 in female CD8 cells compared with males, with similar trends observed at p109 in DP and CD4 Teff subsets and at p110 in CD8 cells (Figure 4C). Taken together, these positional patterns suggest a subtle sex-biased enrichment of hydrophobic amino acids at key TRB CDR3 positions that could be compatible with a slightly increased potential for self-reactivity and cross-reactivity in female CD8 T cells (Stadinski et al., 2016).

### Slight sex-based variations in thymic TCR generation probability distribution

Another approach to evaluating biases in the generation and selection of the TCR repertoire is to compare the probability of generation (Pgen) of CDR3 nucleotide sequences (Figure 1). Pgen represents the likelihood of a sequence being produced, according to models of V(D)J recombination (Murugan et al., 2012; Elhanati et al., 2014). For DP and CD8 cells only and for each TCR chain, a sequence generation model was created using 100,000 non-productive nucleotide sequences. Using this model, the Pgen of all productive TCR sequences that make up each repertoire was calculated.

**Figure 5.**
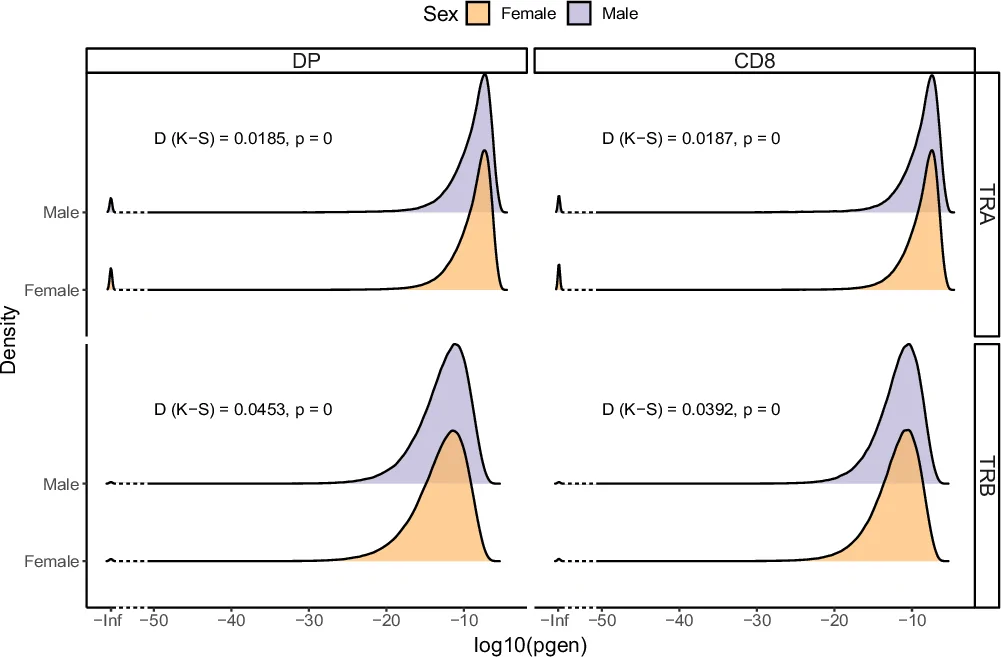
Probabilities of generation of thymic T cell receptors (TCRs) in males and females. The figure shows the distribution of log10 probability of generation (Pgen) values of sequences between males and females for TRA and TRB in double-positive (DP) and CD8 single-positive (SP) thymic T cells. A V(D)J recombination model was created using 100,000 non-productive random sequences derived from the nonproductive sequences of all individuals for each TCR chain, both for DP and CD8 cells (Marcou et al., 2018). These models were then used to estimate the Pgen values of sequences for each individual (Sethna et al., 2019). The overall distribution comparison between males and females was tested using two-sided Kolmogorov-Smirnov tests, with the D value and associated p-value indicated in each panel. Males are depicted in violet and females in orange.

When comparing the distribution of Pgen between males and females, we observe a very slight difference of distribution, characterized by a nearly null Kolmogorov-Smirnov score (D_K-S_ <0.05; Figure 5). This difference is more pronounced in the TRB compared to the TRA chain (Figure 5). As this minor discrepancy might be indicative of variations among individuals irrespective of their sex, we have generated 20,000 permuted mixed-sex groups utilizing our dataset. Our findings revealed a significant distribution difference of Pgen sequences between these mixed groups population (DP – TRA: D_K-S_=0.0183 ± 0.0145 ($\mu \pm sd$), DP – TRB: D_K-S_=0.0190 ± 0.0161, CD8 – TRA: D_K-S_=0.0227 ± 0.0153, and CD8 – TRB: D_K-S_=0.0259 ± 0.0140). However, the observed difference in the Pgen distribution between males and females is higher than that between the mixed groups in the DP TRB.

### Similar network structure of thymic TCR repertoires in males and females

**Figure 6.**
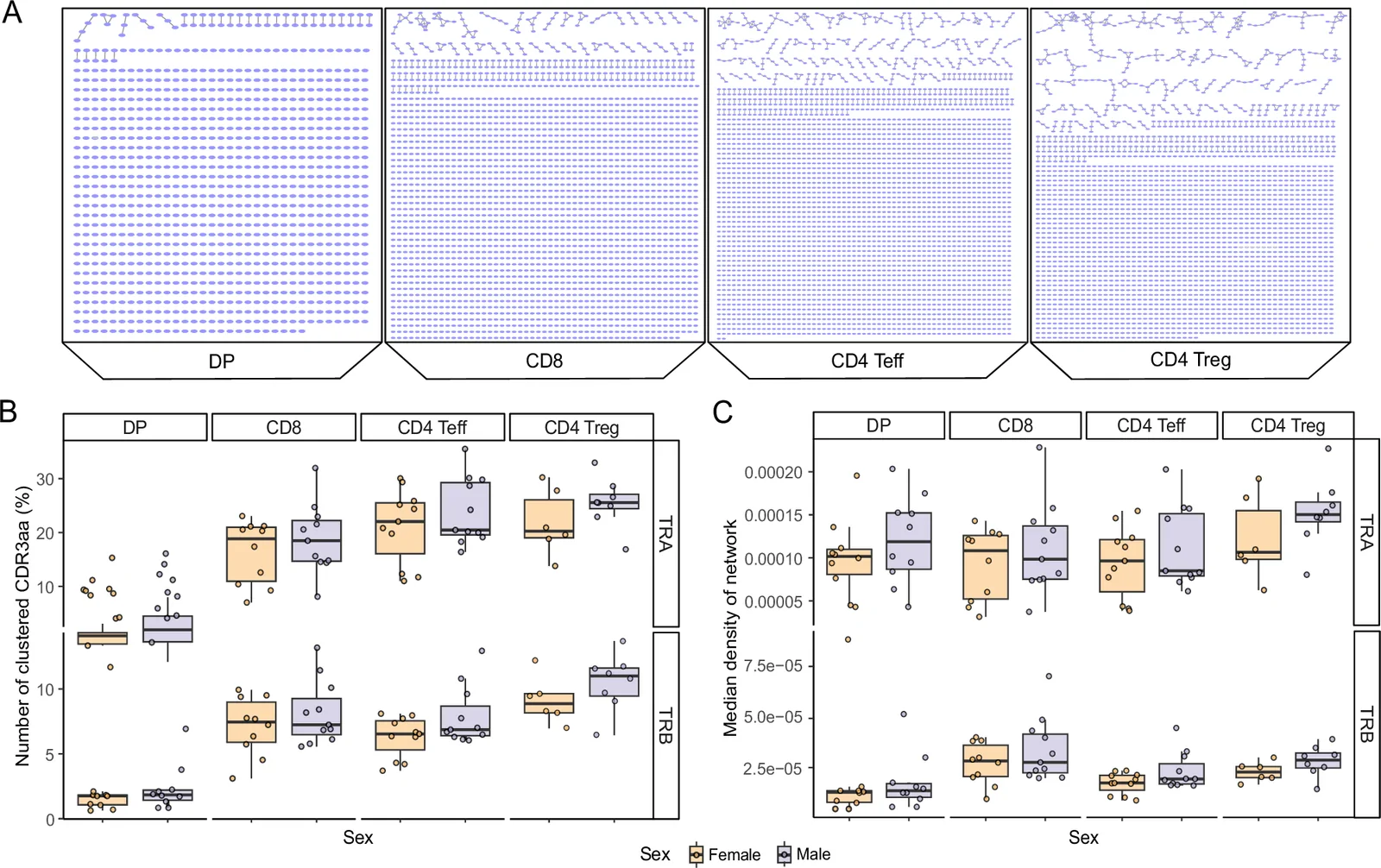
Comparable T cell receptor (TCR) repertoire network structure based on CDR3 amino acid sequence similarity between males and females. For each sample, 100 random subsamplings were performed on the minimum number of CDR3aa per cell subtype. Two CDR3aa are linked if they have a Levenshtein distance of one. (**A**) TRA Network structure of subsampled TCR repertoire of a male subject for double-positive (DP), CD8, CD4 Teff, and CD4 Treg single-positive (SP; from left to right). Each point on the graph represents a CDR3aa. (**B–C**) Comparison of the proportion of linked sequences (**B**) and network density (**C**) between male and female samples, in DP (n=20), CD8 (n=21), CD4 Teff (n=22), and CD4 Treg (n=14). Each point on the graph represents the median value from 100 subsampling iterations for each sample. Statistical analysis using the two-sided Wilcoxon test revealed no significant sex differences for these two metrics (p>0.05). Males are depicted in violet and females in orange.

Next, we evaluated whether thymic selection in males and females is directed towards similar or distinct sequences. For each TCR repertoire, CDR3aa sequences are connected if they differ by one aa, that is have a Levenshtein distance of = 1 (Figure 1). Such linked sequences have a high probability of sharing the same specificities (Meysman et al., 2019). For each TCR chain and thymic T cell subset, CDR3aa repertoires were downsampled to the size of the smallest sample to enable comparisons across samples. This process was repeated 100 times. The downsampling sizes used for each chain and subset are provided in Supplementary file 1. The shapes of the network are depicted in Figure 6A.

To analyze these clusterings with greater precision, we used various metrics. When comparing the number of clustered sequences in each individual network between males and females, no significant differences were observed for any cell type in either chain (Figure 6B). Similarly, the degree of sequence similarity, as indicated by the density of edges between sequences within an individual’s TCR repertoire, was comparable between males and females (Figure 6C). These findings indicate that there are no significant sex-specific differences in the similarity of CDR3aa sequences, suggesting that males and females produce CDR3aa sequences with the same degree of variability within their TCR repertoires.

### Absence of sex-specific CDR3aa motifs in thymic TCR repertoires of males and females

We then sought to determine whether we could detect CDR3aa sequence motifs that were more prevalent in one group compared to the other, in order to evaluate whether the selection of CDR3aa was biased towards particular sequences differently between males and females (Figure 1). To identify sequences with shared characteristics that might indicate a probable common specificity among individuals of the same group, the analysis focused exclusively on CDR3aa sequences of the TRB, as this region has been shown to have a greater influence on TCR specificity (Springer et al., 2021; Korpela et al., 2023). Local motifs were defined using Gliph2 (Huang et al., 2020), which are sequences of three to five aa, as well as global motifs, which are sequences of more than three aa where, at one position, an aa can be substituted by another aa if it has a positive BLOSUM62 score (Henikoff and Henikoff, 1992). Importantly, only motifs containing public CDR3aa sequences shared by at least two individuals were retained, thereby excluding private or individual-specific sequences from the analysis.

**Figure 7.**
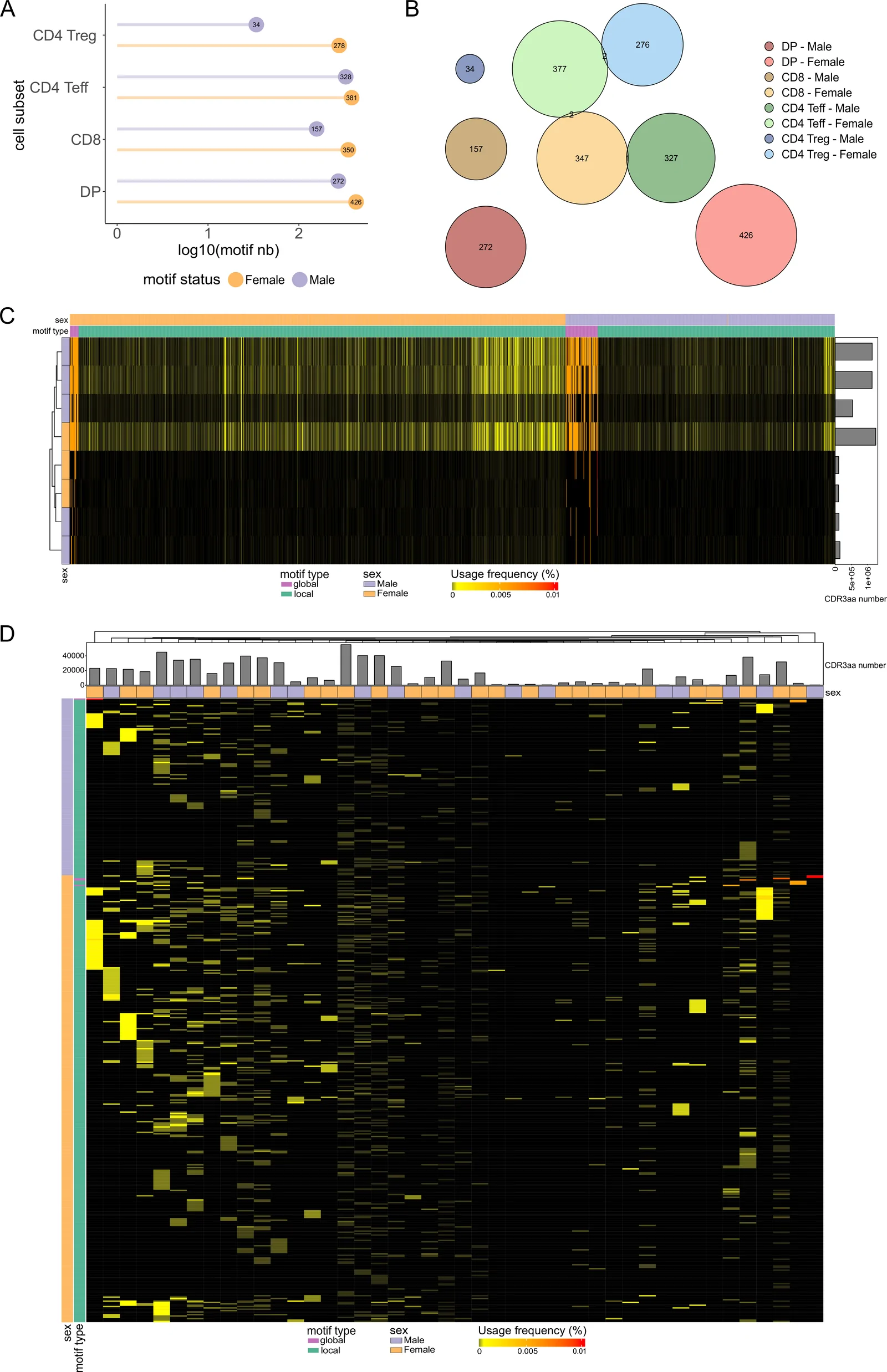
Thymic TRB T cell receptor (TCR) sex-associated motifs. Different structural motifs found differentially expressed between males and females in our dataset. We distinguish local motifs as distinct aa sequences, and global motifs as motif regions with one variable aa position maintaining a BLOSUM62 score of ≥0. (**A**) Number of male and female associated motifs by cell subset. (**B**) Euler diagram illustrating the distribution and overlap between all sex-associated motifs. The numbers indicate the number of motifs in overlap zones. (**C–D**) Validation of these sex-associated TRB CDR3aa motifs. The following heatmap illustrates the usage of all the TRB CDR3aa motifs in the external thymic pediatric dataset (Heikkilä et al., 2021; Mattila et al., 2023) (**C**) and those of TRB CD8 in the peripheral dataset (**D**). Sex and total CDR3aa number are depicted by sample. Males are depicted in violet and females in orange, then local motifs in blue-green and global motifs in magenta.

We identified several hundred motifs that were differentially expressed between males and females for each cell subpopulation. A greater number of motifs were specific to females, ranging from 426 motifs for DP to 278 motifs for CD4 Tregs, compared to males, ranging from 328 motifs for CD4 Teffs to 34 motifs for CD4 Tregs (Figure 7A). The identified motifs are mainly local motifs and largely confined to their respective sex group, indicating that motifs identified in females are predominantly found in females, and vice versa (Figure 7—figure supplements 1–4).

**Figure 7—figure supplement 1.**
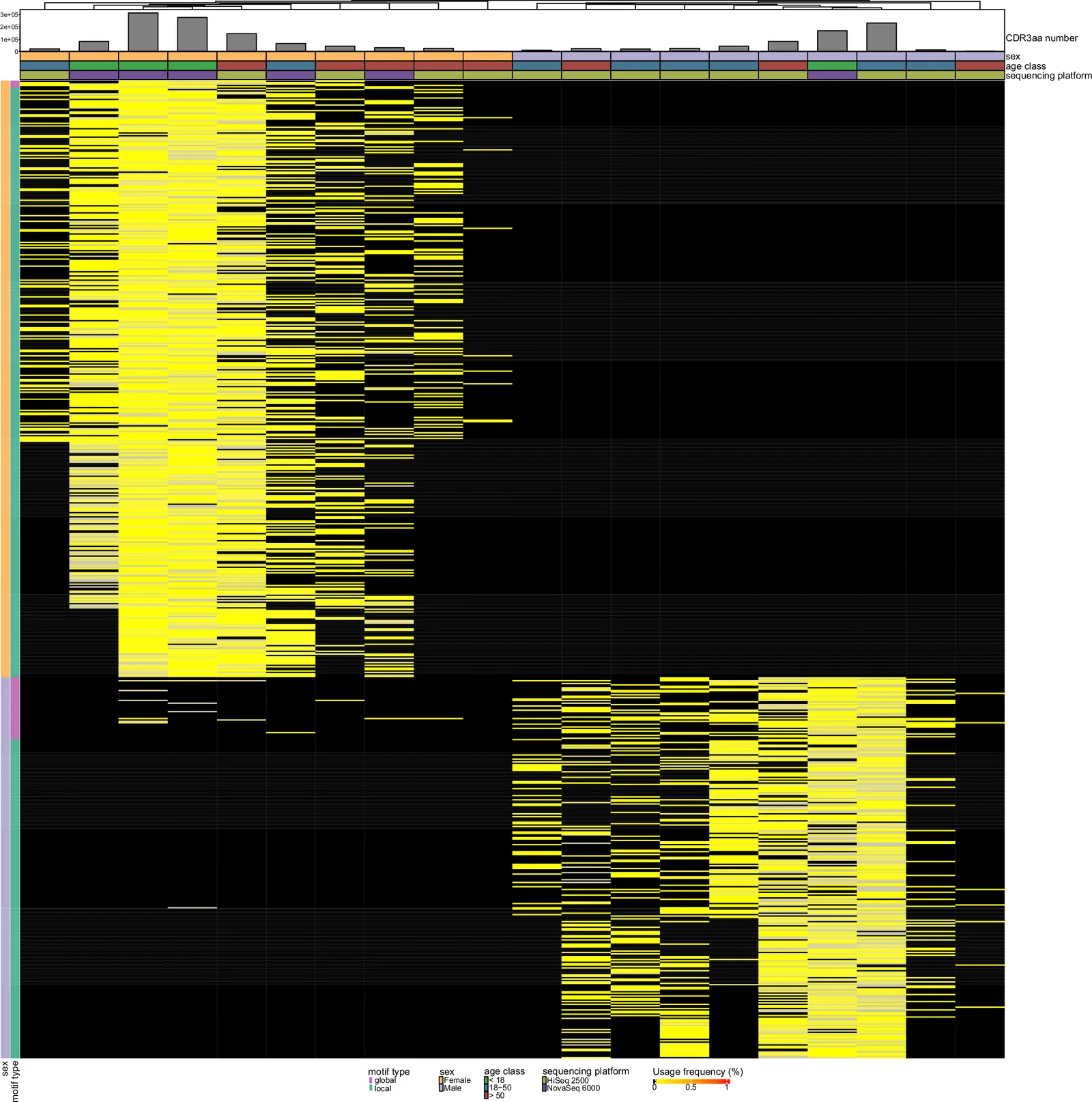
Double-positive (DP) TRB thymic sex-associated motifs in our dataset. Different structural motifs in the CDR3 amino acid (CDR3aa) region found differentially expressed between males and females within our dataset. Local motifs refer to distinct amino acid sequences, whereas global motifs represent motif regions with a single variable amino acid position maintaining a BLOSUM62 score ≥0. The heatmap showcases the differential usage of TRB CDR3aa motifs between males and females in DP cells. Hierarchical clustering reveals clear segregation of individuals by sex, with most CDR3aa motifs being almost exclusively expressed in one sex while absent in the other. Sample attributes are visualized as follows: (1) Sex: males in violet, females in orange; (2) Age class: children (<18 years) in green, young adults (18–50 years) in blue, older adults (>50 years) in red; (3) sequencing platform: HiSeq 2500 samples in persimmon, NovaSeq 6000 samples in purple; (4) the total CDR3aa number. Motifs overexpressed in males are shown in violet, those overexpressed in females in orange; local motifs are indicated in blue-green and global motifs in magenta.

**Figure 7—figure supplement 2.**
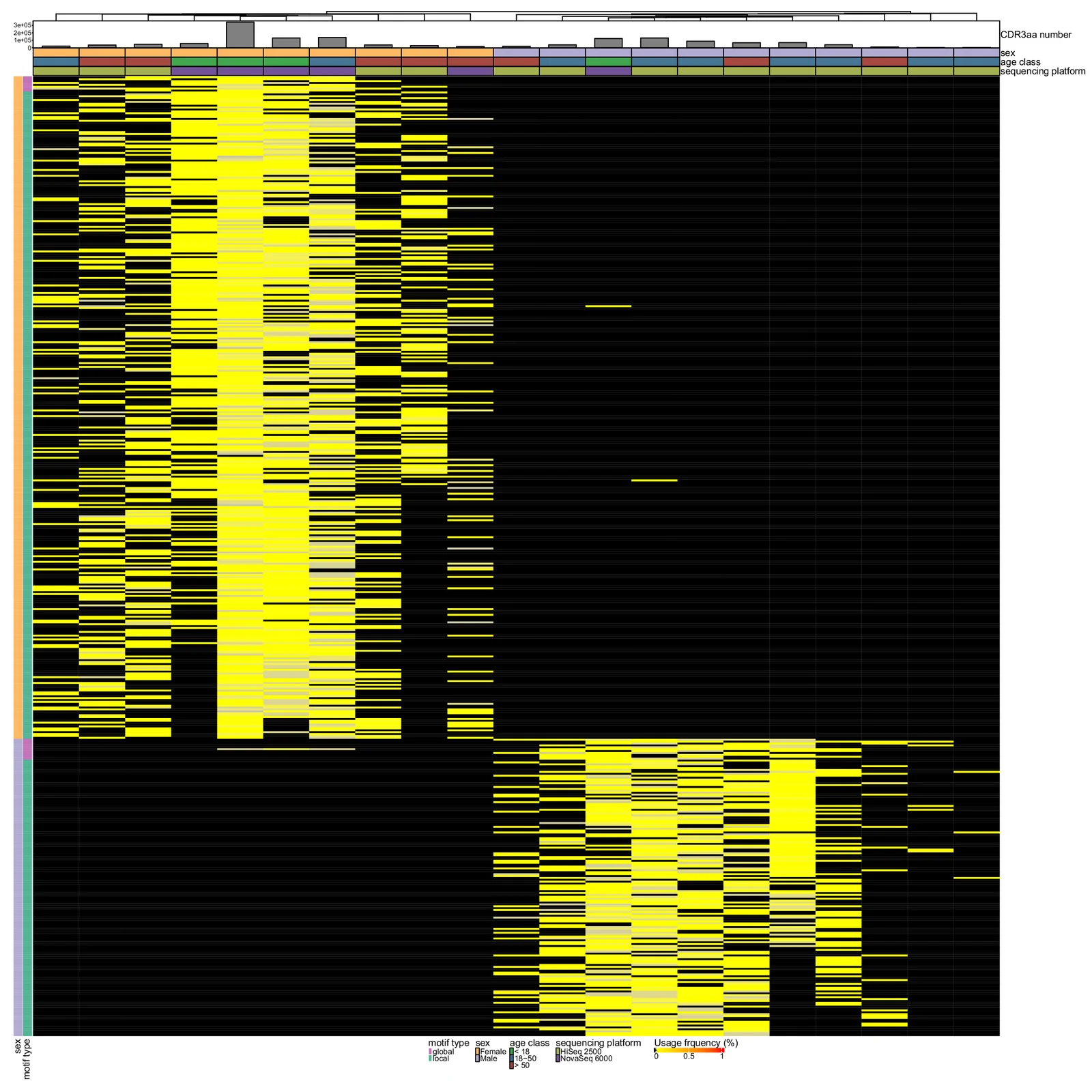
CD8 TRB thymic sex-associated motifs in our dataset. Different structural motifs in the CDR3 amino acid (CDR3aa) region found differentially expressed between males and females within our dataset. Local motifs refer to distinct amino acid sequences, whereas global motifs represent motif regions with a single variable amino acid position maintaining a BLOSUM62 score ≥0. The heatmap showcases the differential usage of TRB CDR3aa motifs between males and females in CD8 cells. Hierarchical clustering reveals clear segregation of individuals by sex, with most CDR3aa motifs being almost exclusively expressed in one sex while absent in the other. Sample attributes are visualized as follows: (1) Sex: males in violet, females in orange; (2) Age class: children (<18 years) in green, young adults (18–50 years) in blue, older adults (>50 years) in red; (3) sequencing platform: HiSeq 2500 samples in persimmon, NovaSeq 6000 samples in purple; (4) the total CDR3aa number. Motifs overexpressed in males are shown in violet, those overexpressed in females in orange; local motifs are indicated in blue-green and global motifs in magenta.

**Figure 7—figure supplement 3.**
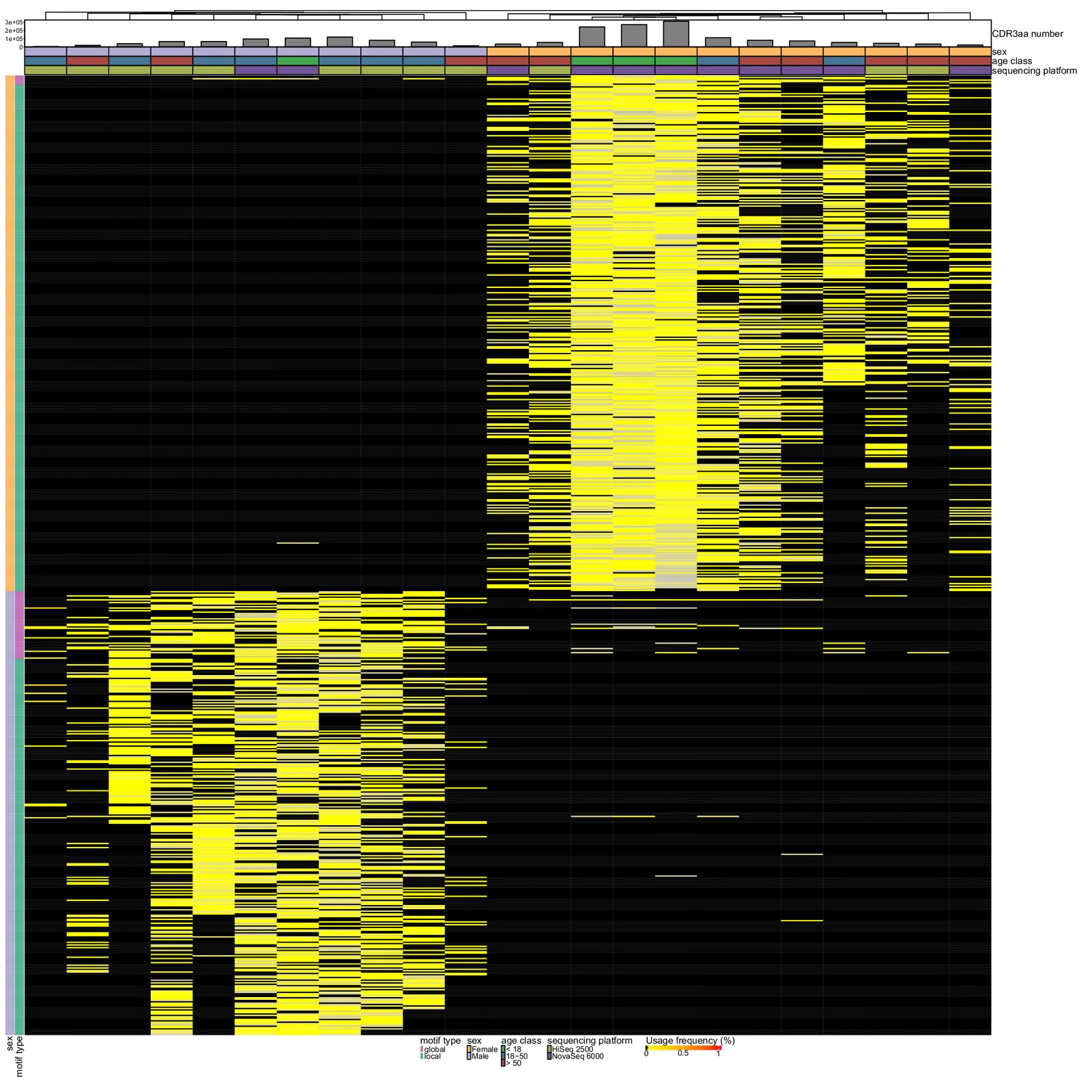
CD4 Teff TRB thymic sex-associated motifs in our dataset. Different structural motifs in the CDR3 amino acid (CDR3aa) region found differentially expressed between males and females within our dataset. Local motifs refer to distinct amino acid sequences, whereas global motifs represent motif regions with a single variable amino acid position maintaining a BLOSUM62 score ≥0. The heatmap showcases the differential usage of TRB CDR3aa motifs between males and females in CD4 Teff cells. Hierarchical clustering reveals clear segregation of individuals by sex, with most CDR3aa motifs being almost exclusively expressed in one sex while absent in the other. Sample attributes are visualized as follows: (1) Sex: males in violet, females in orange; (2) Age class: children (<18 years) in green, young adults (18–50 years) in blue, older adults (>50 years) in red; (3) sequencing platform: HiSeq 2500 samples in persimmon, NovaSeq 6000 samples in purple; (4) the total CDR3aa number. Motifs overexpressed in males are shown in violet, those overexpressed in females in orange; local motifs are indicated in blue-green and global motifs in magenta.

**Figure 7—figure supplement 4.**
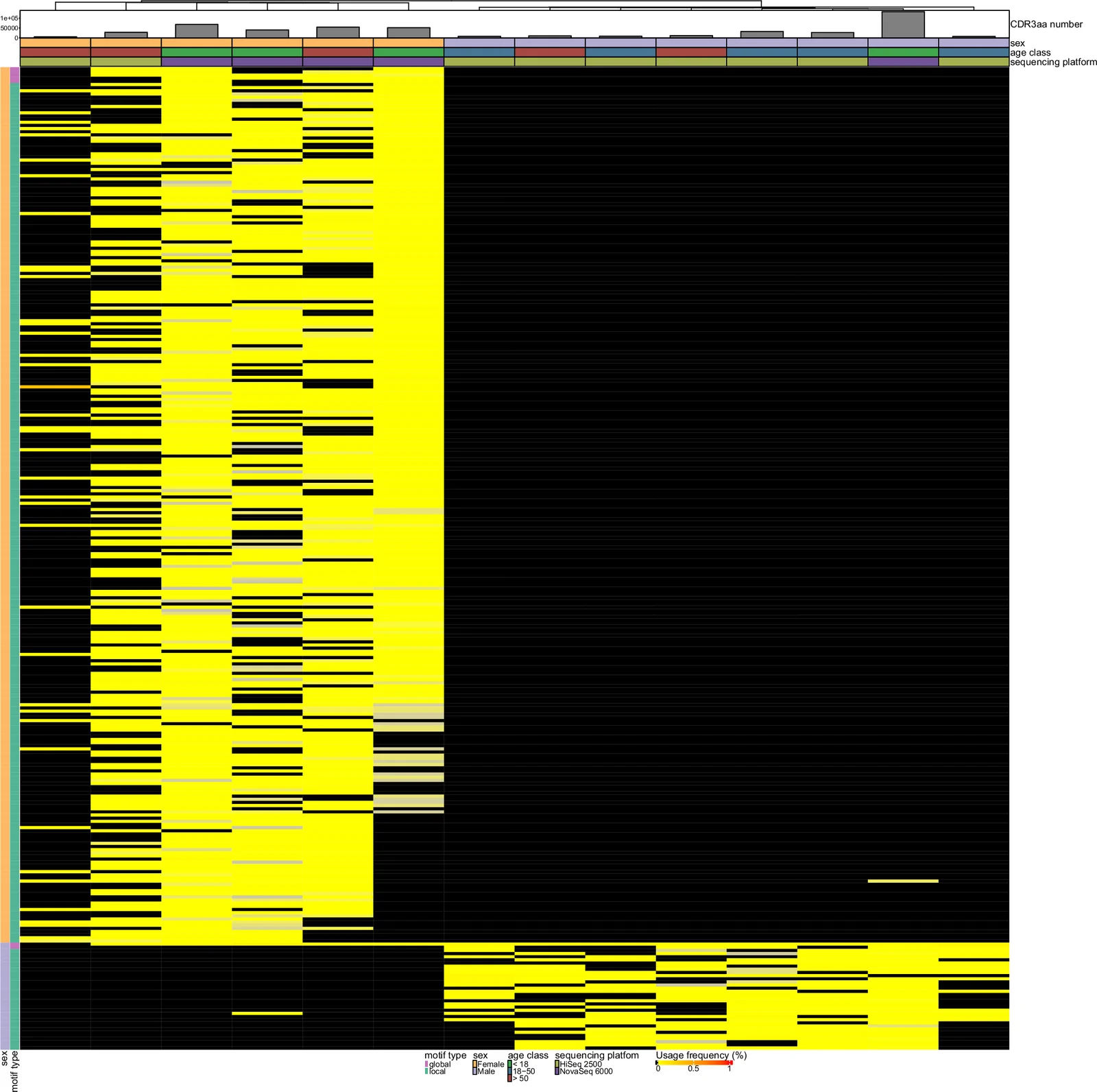
CD4 Treg TRB thymic sex-associated motifs in our dataset. Different structural motifs in the CDR3 amino acid (CDR3aa) region found differentially expressed between males and females within our dataset. Local motifs refer to distinct amino acid sequences, whereas global motifs represent motif regions with a single variable amino acid position maintaining a BLOSUM62 score ≥0. The heatmap showcases the differential usage of TRB CDR3aa motifs between males and females in CD4 Treg cells. Hierarchical clustering reveals clear segregation of individuals by sex, with most CDR3aa motifs being almost exclusively expressed in one sex while absent in the other. Sample attributes are visualized as follows: (1) Sex: males in violet, females in orange; (2) Age class: children (<18 years) in green, young adults (18–50 years) in blue, older adults (>50 years) in red; (3) sequencing platform: HiSeq 2500 samples in persimmon, NovaSeq 6000 samples in purple; (4) the total CDR3aa number. Motifs overexpressed in males are shown in violet, those overexpressed in females in orange; local motifs are indicated in blue-green and global motifs in magenta.

**Figure 7—figure supplement 5.**
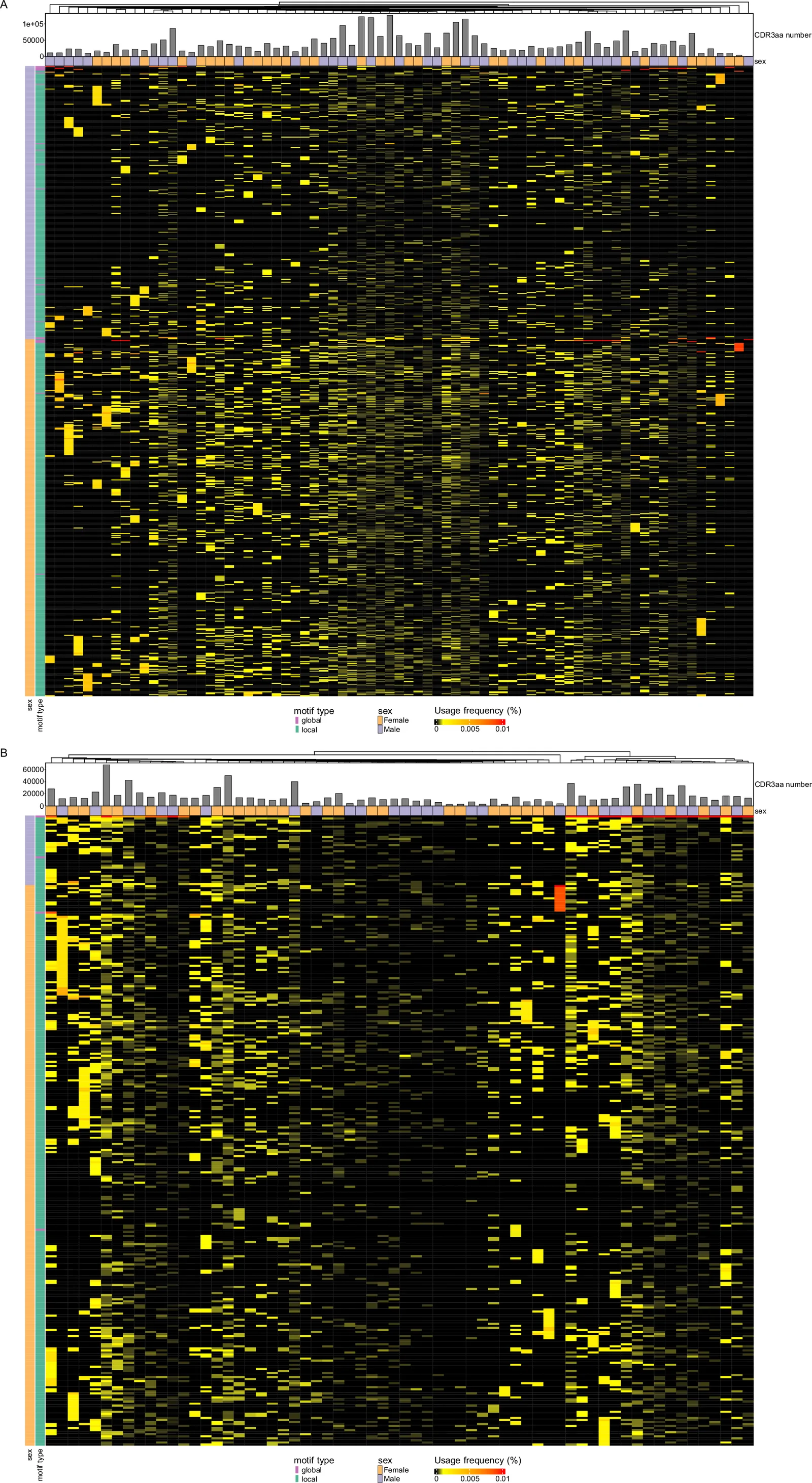
TRB thymic sex-associated motifs are not differentially expressed between males and females in peripheral T cell receptor (TCR) repertoire. Heatmap representing TRB CDR3aa motif usage differentially expressed between males and females in CD4 Teff (n=75; n_males = 38, n_females = 37) (**A**) and CD4 Treg (n=64; n_males = 29, n_females = 35) (**B**) cells in a peripheral TCR repertoire dataset. Sex and total CDR3aa number are depicted by samples. Males are depicted in violet and females in orange and local motif in blue-green and global motifs in magenta.

As illustrated in Figure 7B, we observed minimal motif overlap between different cell subtypes. Only two female-associated motifs overlapped: one between CD4 Teffs and CD4 Tregs, and another between CD4 Teffs and CD8 cells (Figure 7B). In contrast, no overlap was found between male-associated motifs (Figure 7B). Furthermore, it was observed that there was an overlap between one motif associated with female CD8 cells and a motif associated with male CD4 Teff cells (Figure 7B).

We then proceeded to evaluate the usage of the differentially expressed motifs in external datasets. There is no publicly available thymic TCR dataset that reports repertoires according to cell subsets. We thus tested these motifs on TCRs from pediatric bulk thymocytes, which contain TCR data from children aged between two and eight months, with a male-to-female ratio of 5:3 (Heikkilä et al., 2021; Mattila et al., 2023). We calculated the usage of these differentially expressed motifs in each sample of this dataset. We were unable to separate individuals by sex using these motif usages (Figure 7C).

We then evaluated these motifs using a peripheral blood TCR dataset from healthy individuals, where CD8, CD4 Teff, and CD4 Treg cells had been sorted. The usage of these motifs could not distinguish males from females across all cell subsets (Figure 7D, Figure 7—figure supplement 5).

### Sex-biased enrichment of TCRs associated with autoimmune diseases and bacterial antigens

We sought to identify the usage of TCRs with known specificity as represented in the IEDB, Mc-PAS, and VDJdb public databases (Vita et al., 2019; Tickotsky et al., 2017; Shugay et al., 2018). We compiled the TCRs from these three databases, retaining only those with high sequence reliability scores (see Materials and Methods). We focused on TRB sequences exclusively, due to their greater representation in the databases (Springer et al., 2021; Rosati et al., 2017). We identified 55,368 unique TRB CDR3aa sequences with high reliability and specificity assignment scores for at least one specificity.

We classified the TCR specificities based on the antigen they recognize: viral or bacterial peptides, or human peptides overexpressed in cancers, AIDs, or neither of these diseases (Figure 8—figure supplement 1A). We sought to identify the TRB CDR3aa sequences in our thymocyte dataset. Depending on the individuals and cell subtypes, they represented between 0.82% and 3.58% of the TRB repertoires.

**Figure 8.**
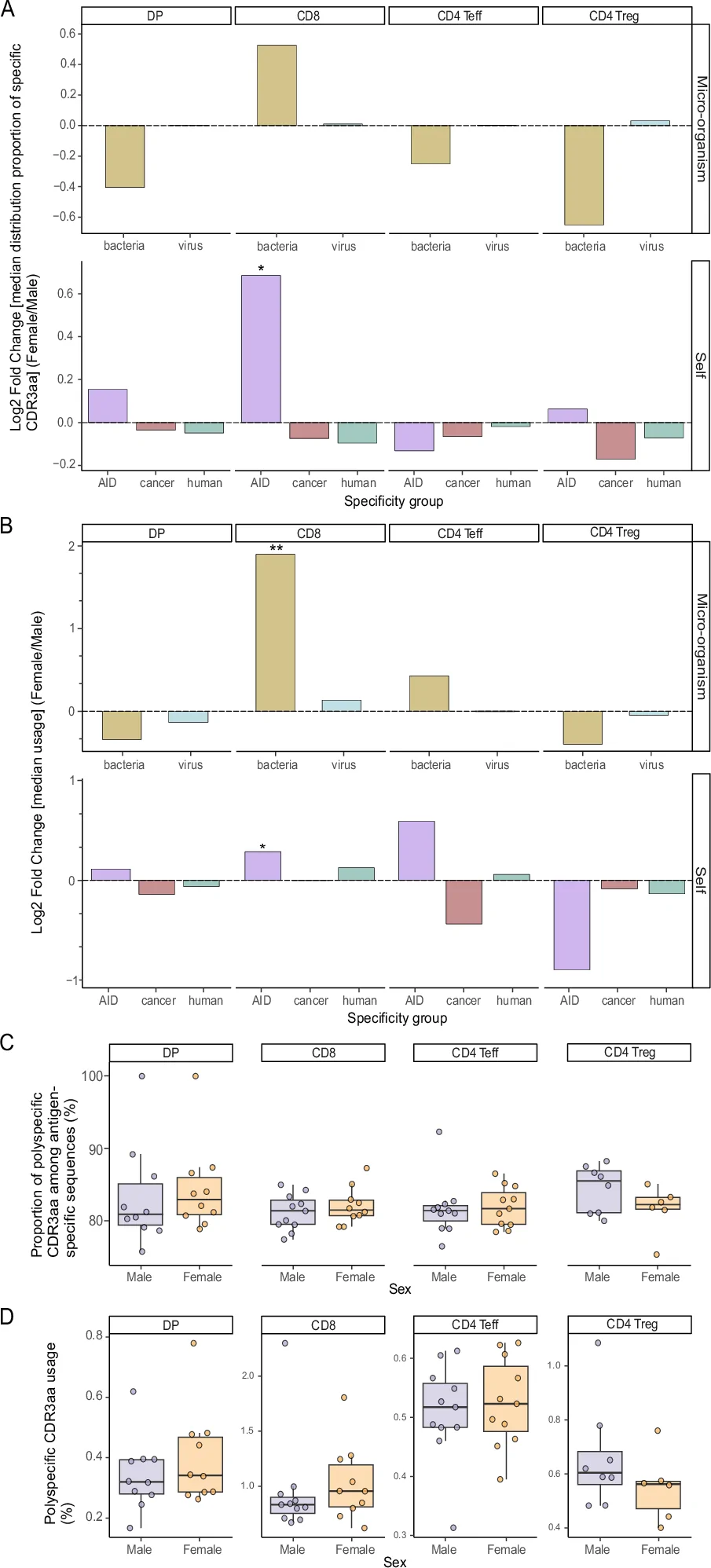
Sex-biased enrichment of T cell receptors (TCRs) specific for known antigens. From a pooled and curated database, an exact match with this database infers the specificity of TRB CDR3aa of our thymic dataset. Many specificity groups are defined according to the nature of the antigen peptide targeted (bacteria, virus, autoimmune disease [AID], cancer, and self-peptide not associated with disease [human]). This analysis compares the distributions of the proportion of unique TRB CDR3aa sequences with a specific specificity (**A**) and their usage (**B**) between females and males across cell subtypes, using the log2 fold change of the median values (females over males), following each specificity group, in double-positive (DP; n=20), CD8 (n=21), CD4 Teff (n=22), and CD4 Treg (n=14) cells. These groups of specificity are additionally classified as microorganisms in the top section (bacteria in gold and virus in light blue) and self at the bottom (AID in magenta, cancer in red, human in blue-green). Polyspecific CDR3aa are defined here as CDR3aa capable of recognizing multiple antigens from different organisms (for no self-antigens) or from different specificity groups (e.g. categorized microorganisms, categorized self-antigens, allergens…). The proportion of polyspecific CDR3aa among antigen-specific sequences (**C**) and their usage (**D**) is compared between males and females, in double-positive (DP; n=20), CD8 (n=21), CD4 Teff (n=22), and CD4 Treg (n=14) cells. Asterisks indicate significant differences between males and females based on the two-sided Wilcoxon test p-value (*: p<0.05, **: p<0.01). Males are depicted in violet and females in orange.

We observed that unique TRB CDR3aa sequences specific to self-antigens not associated with pathologies and those associated with cancers are proportionally more represented in our thymic TCR dataset than in the pooled reference database, in both females and males (Figure 8—figure supplement 1B). This pattern was consistent across all cell subtypes (Figure 8—figure supplement 1B). These comparisons to the reference database are presented for descriptive purposes only, as differences between an experimental thymic repertoire and a curated database are expected given the structure of the reference resource. We then compared the distribution of these CDR3aa between males and females for each cell subtype. Strikingly, in the TCR repertoires with one specificity only, we observed a significantly higher proportion of unique TRB CDR3aa sequences associated with AIDs in female CD8 SP cells compared to male CD8 SP cells (Figure 8A). Furthermore, in the specific TCR repertoires only, there was a trend towards a higher proportion of thymic sequences with specificity associated with bacterial compounds in female CD8 SP cells compared to males, and an opposite trend was observed for CD4 Tregs SP (Figure 8A). These differences observed in CD8 SP cells persisted when examining the usage of these sequences (i.e. cumulative usage frequency), with significantly higher usage of sequences with specificity associated with bacterial compounds and those associated with AIDs in females compared to males (Figure 8B). Of particular note are the donor-resolved analyses (Figure 8—figure supplements 1B and 2), in which each individual is represented separately. These confirm that these patterns are not driven by a single donor.

**Figure 8—figure supplement 1.**
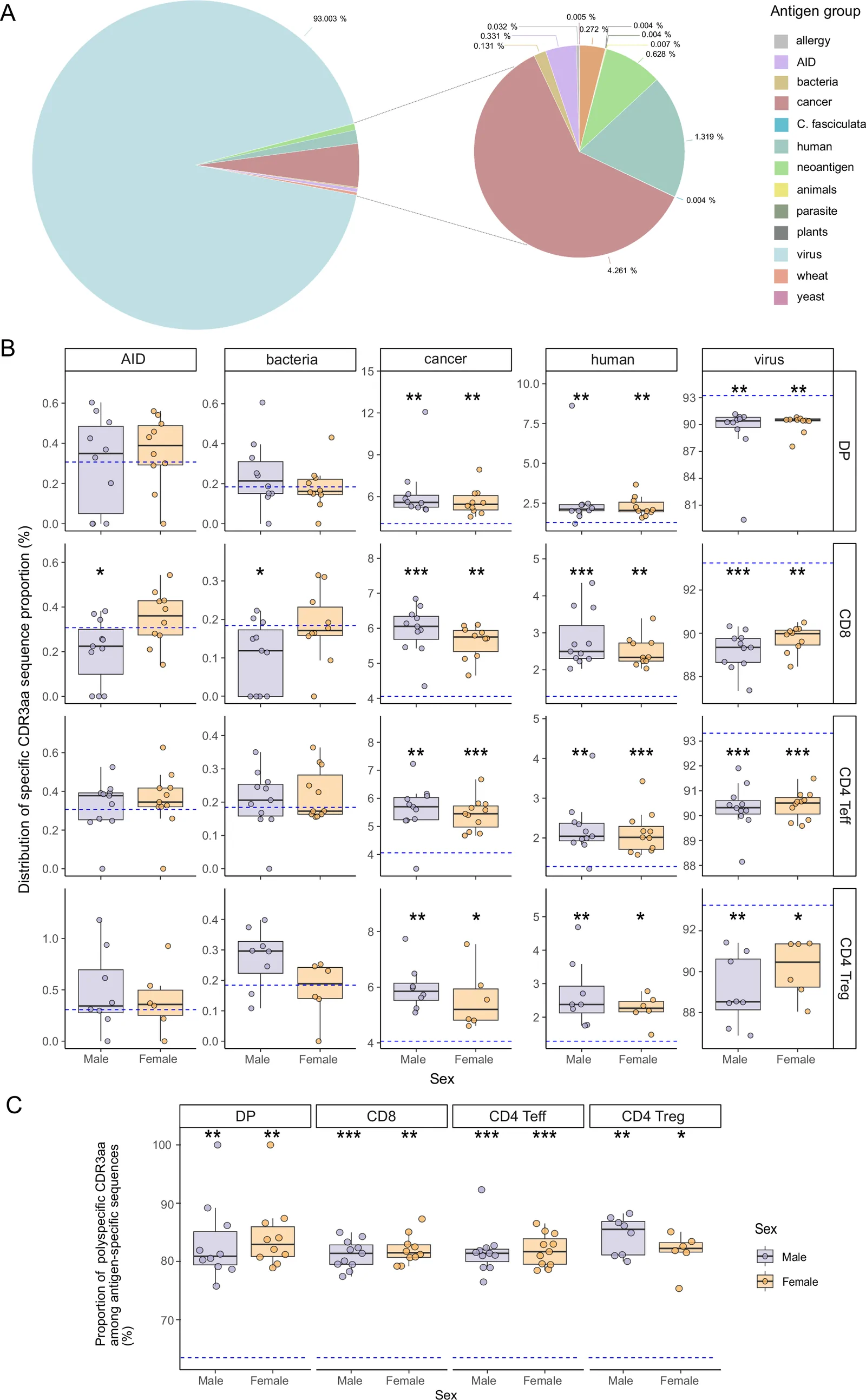
Enrichment of cancer-associated, unmodified self-peptide specific, and polyspecific T cell receptor (TCR), in our thymic dataset compared to the initial curated database. (**A**) Representation of the specificity groups in the pooled and curated database. (**B**) Proportion of unique CDR3aa TRB in the specific TCR repertoire for each sample for autoimmune disease (AID), bacteria, cancer, human, and virus groups is compared between males and females in double-positive (DP; n=20), single-positive (SP) CD8 (n=21), SP CD4 Teff (n=22), and SP CD4 Treg (n=14). (**C**) Proportion of polyspecific CDR3aa in the specific repertoire of samples is compared between males and females in DP (n=20), SP CD8 (n=21), SP CD4 Teff (n=22), and SP CD4 Treg (n=14). Asterisks indicate significant differences males or females with the pooled and curated database based on the two-sided Wilcoxon test p-value (*: p<0.05, **: p<0.01). Males are depicted in violet and females in orange.

**Figure 8—figure supplement 2.**
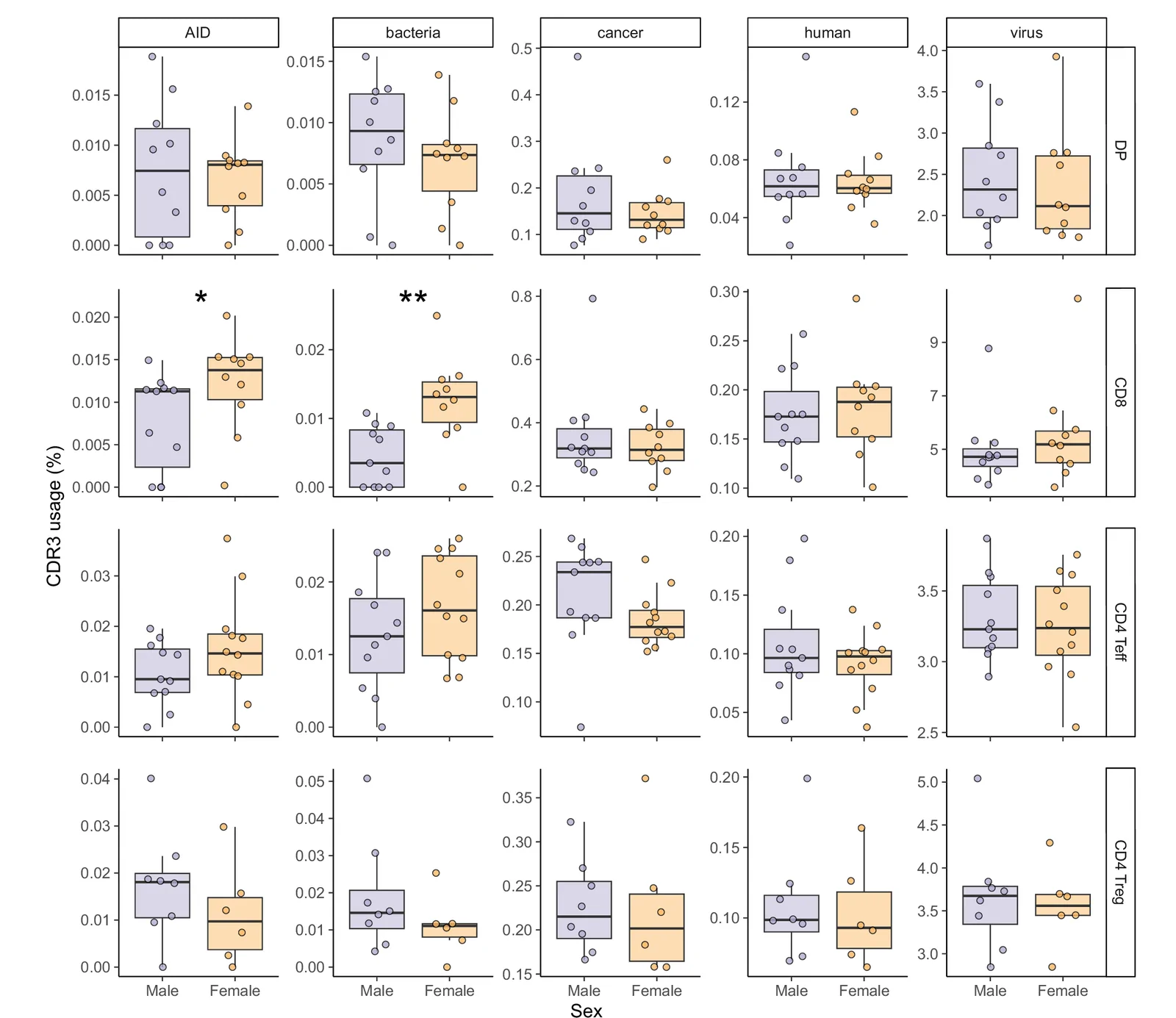
Enrichment of autoimmune disease (AID)-associated T cell receptor (TCR) and bacteria-targeted TCR in CD8 female cells. CDR3aa usage of CDR3aa with a specificity in autoimmunity disease, bacteria, cancer, human, and virus groups, in double-positive (DP; n=20), single-positive (SP) CD8 (n=21), SP CD4 Teff (n=22), and SP CD4 Treg (n=14). Asterisks indicate significant differences between males and females based on the two-sided Wilcoxon test p-value (*: p<0.05, **: p<0.01). Males are depicted in violet and females in orange.

**Figure 8—figure supplement 3.**
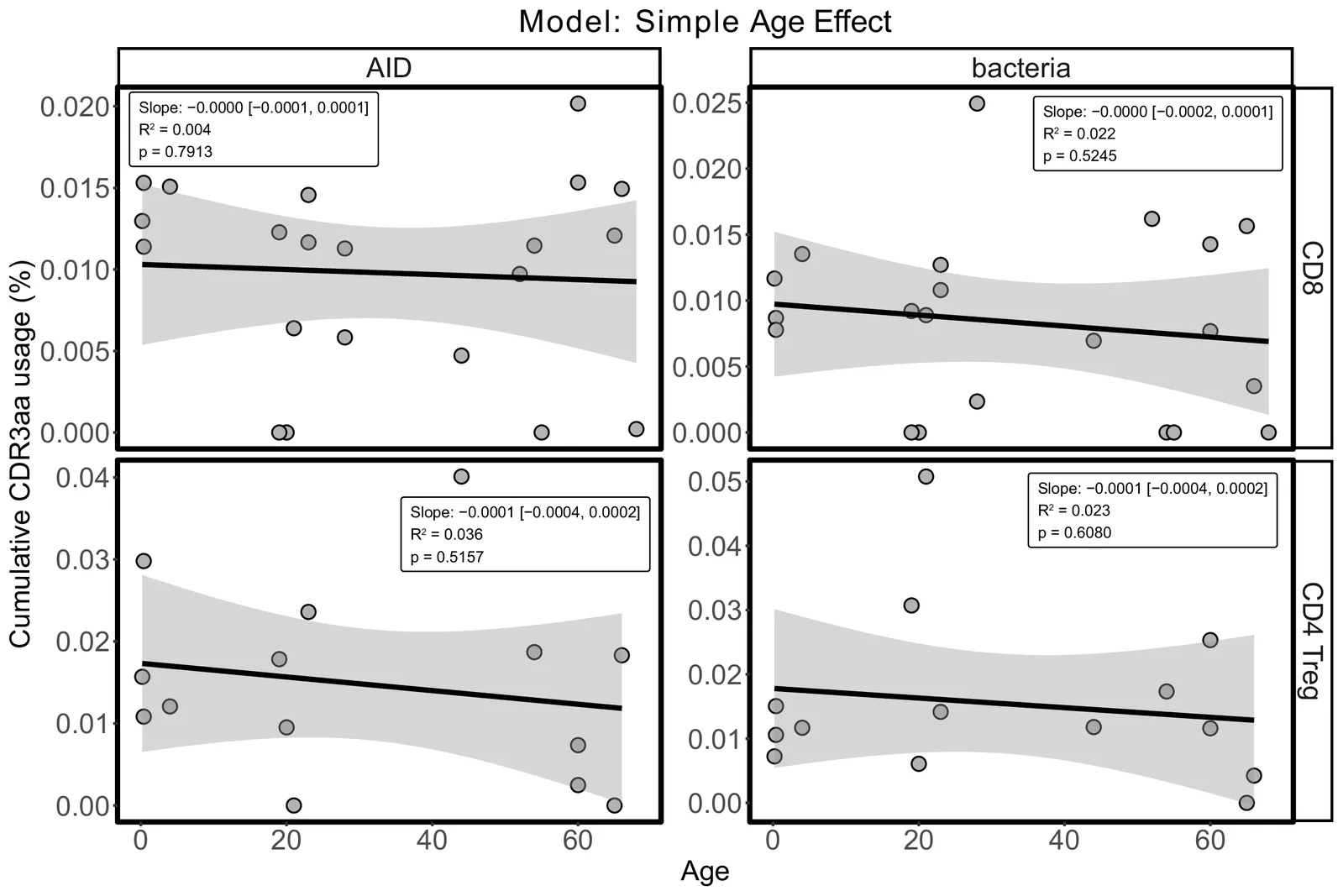
No association between age and thymic usage of CDR3 sequences specific to autoimmunity or bacterial antigens. Linear regression models assessing the effect of donor age on the cumulative usage (%) of TRB CDR3aa sequences with known specificity to self-antigens associated with autoimmunity (AID, left panels) or bacterial antigens (right panels), in CD8 single-positive (SP; top, n=21) and CD4 Treg SP (bottom, n=14) thymic subsets. Each dot represents one donor. The shaded area indicates the 95% confidence interval around the regression line. Reported slope, 95% confidence interval, R², and p-values show no significant effect of age.

Donor age had no significant effect on the sex-biased enrichment of self- and bacteria-specific TRB CDR3 sequences in CD8 SP and CD4 Treg SP cells (p ≥ 0.52), indicating that the observed differences are age-independent (Figure 8—figure supplement 3). In addition, we investigated for the presence of polyspecific TCRs, which are capable of recognizing multiple antigens from different organisms (Wooldridge et al., 2012; Hadrup et al., 2009; Quiniou et al., 2023). These sequences were found to be enriched across all cell subtypes, in comparison to their representation in the reference database (Figure 8—figure supplement 1C). However, no significant differences were observed in the proportion or usage of these polyspecific sequences between males and females. Interestingly, an inverse usage trend was observed between CD8 and CD4 Treg SP cells: females exhibited higher usage of these sequences in the CD8 SP repertoire but a lower usage in the CD4 Treg SP repertoire compared to males (Figure 8C and D).

In conclusion, our analysis of TCR specificity has identified sex-specific differences in the thymic selection of CD8 effector T cells associated with autoimmune and bacterial antigens that are overrepresented in females versus males.

## Discussion

It is noteworthy that there is a paucity of research on potential differences in the TCR repertoire between the sexes, despite the much higher prevalence of AIDs in females, a topic that remains to be fully elucidated. To explore the hypothesis that the generation and/or the selection of the TCR repertoires may be different between sexes requires, it is necessary to study the TCR repertoire where they are formed and selected, in the thymus. We thus generated a unique and valuable dataset comprising thymic samples collected from deceased organ donors of various ages, and young children who have undergone cardiac surgery. Firstly, we separated the various thymocyte populations in order to study both TCR repertoire generation at the level of DP cells, and repertoire selection at the level of CD8, CD4 Teff, and Treg SP cells. We performed bulk sequencing of the TCRs from these cells, as this is the only method that yet generates enough sequences for sensitive comparison at this time. Single-cell sequencing is an analyzing technique that is limited in its scope. It is only capable of analyzing a few thousand cells and would not have enabled the analyses that can be performed with hundreds of thousands of cells.

### TCR generation

Our analyses initially revealed similarities rather than dissimilarities in TCR generation. Our detailed analyses revealed no significant sex differences in relation to the majority of the examined variables when comparing males and females DP cells’ repertoires, except for differences in the usage of a small number of V and J genes from both TCR chains. However, these minor discrepancies are likely attributable to random variation, considering the extensive number of comparisons conducted. This finding is further supported by the observation that there are no significant differences in the usage of TRBV-TRBJ and TRAV-TRAJ gene associations between males and females. We also observed no differences in diversity indexes in DP cells’ repertories between males and females. Altogether, the overall combinatorial diversity of the TCR repertoire remains comparable between sexes.

A detailed analysis of the CDR3aa region in DP cells showed that some hydrophobic aa in the FG loop region are differently found for both TCR chains. However, no significant sex-based differences were found in the usage of hydrophobic aa at the critical p109 and p110 positions in TRB that have been described as influencing self-antigen recognition due to the impact of hydrophobic aa on TCR self-reactivity (Khosravi-Maharlooei et al., 2019; Lu et al., 2019; Stadinski et al., 2016). These findings suggest that the subtle sex-based differences in the FG loop’s aa usage are minor and do not significantly impact the overall characteristics of the CDR3 region in a sex-dependent manner.

The Pgen analysis of DP cells revealed only minimal differences in the distribution of Pgen between males and females, which were similar to those observed in control groups. This indicates that the mechanisms governing TCR generation are largely consistent across sexes, with only minor, likely random, deviations.

Additionally, although we identified TRB CDR3aa sequence motifs that were differentially expressed between males and females at the DP cell stage in our dataset, these motifs could not reliably differentiate individuals by sex in external datasets. This indicates that they are more dataset-specific than sex-specific. This issue may also be explained by the different characteristics of our data and that of the other dataset. For example, in the Arstila dataset, the analyses were performed on unsorted cells from young subjects. Younger individuals tend to have a more diverse and richer TCR repertoire, with greater repertoire sharing in the periphery, which could potentially obscure sex-specific patterns (Trofimov et al., 2022; van de Sandt et al., 2023). This suggests that these motifs might be context-specific or influenced by other factors such as age, genetic background, or environmental exposures. Moreover, we did not observe any sex-specific differences in CDR3 TRB specificity usage in DP cells’ repertoires. In summary, our results from DP cells analyses could not identify any relevant sex differences in TCR generation.

### TCR selection

We identified three genes that show differential usage between males and females. While it is possible that some of these differences are merely random, resulting from multiple comparisons, the preferential use of TRBV6-5 in females has previously been observed in the peripheral TCR repertoire (Schneider-Hohendorf et al., 2018). Notably, this gene is overrepresented in female lupus patients compared to their controls (Roca et al., 2019; Hou et al., 2023). Although the preferential usage of TRBV6-5 in females is consistent with previous peripheral observations, external validation of this thymic bias remains challenging. Publicly available TCR datasets rarely provide (i) sorted thymocyte subsets, (ii) balanced sex representation, and (iii) sufficient sequencing depth within each subset to allow reliable comparison of gene usage patterns. Moreover, differences in tissue origin (peripheral blood versus thymus), age distribution, and technical protocols may substantially influence TRBV usage frequencies. For these reasons, direct replication in independent thymic cohorts is currently not feasible. Our permutation-based internal control analysis was therefore implemented to ensure that the observed signal was not attributable to random donor grouping or individual-level variation.

Our most striking observation relates to significant sex-specific differences in inferred specificities of CD8 T cells, with a biased CD8 selection process in the thymus favoring sequences associated with AIDs and bacterial antigens in females. The fact that this bias does not affect DP cells indicates that it is related to TCR selection rather than generation. Its relevance is strongly supported by the fact that (i) there is no such bias for other self-antigens that are not associated with autoimmunity and (ii) the bias affects both the effectors and regulators of autoimmunity with a directionality compatible with the increased prevalence of AID in females. TCRs associated with autoimmunity-related self-antigens have been observed to be increased in CD8 effector T cells, while decreased levels have been seen in CD4 Tregs. It should be noted that this bias also affects certain bacterial antigens, although not viral antigens. This phenomenon could be explained by the fact that bacterial antigens express numerous mimotopes of self-antigens and are known to contribute to the shaping of the TCR repertoire. The observed bias towards bacterial antigens in female CD8 T cells could also provide more effector cells specific for bacterial antigens that mimic self-antigens, contributing to AID development by molecular mimicry (Rojas et al., 2018). In this line, antigens linked to celiac disease (CeD) and type 1 diabetes (T1D) are highly represented in the database and these two AIDs have been linked to bacterial components that may mimic self-antigens (Repac et al., 2023; Girdhar et al., 2022; Petersen et al., 2020; Vazquez et al., 2021; Tai et al., 2016). As is well-established, the gut microbiota influences thymic development of T cells (Hebbandi Nanjundappa et al., 2022). Sexual hormones could influence this biased selection by affecting the composition of the gut microbiota (Markle et al., 2013) and/or by affecting the selection of the TCRs specific for these antigens. In addition to these specific patterns, we observed a significant increase in the usage of hydrophobic amino acids at the central p109 position of the TRB CDR3aa in female CD8 thymocytes, with a similar trend at p110. Hydrophobic residues at these positions have been implicated in enhanced self-reactivity and cross-reactivity of CD8 T cells (Khosravi-Maharlooei et al., 2019; Lu et al., 2019; Stadinski et al., 2016), suggesting that female CD8 thymocytes may be slightly enriched for TCRs with intrinsically higher potential for recognizing self-like or cross-reactive peptide MHC complexes. It is noteworthy that the positional hydrophobicity of CD8 T cells CDR3s is consistent with the over-representation of sequences annotated as recognizing autoimmune-associated self-antigens, pointing to a more autoreactive TCR repertoire.

Finally, we have recently described polyspecific TCRs that can respond to multiple unrelated viral antigens and hypothesized their possible involvement in autoimmunity (Quiniou et al., 2023). We defined a CDR3 as being polyspecific if the TCRs containing it have been experimentally associated with epitopes from at least two different antigen origins. We observed a higher usage of polyspecific sequences in the CD8 SP repertoire of females, although this was not statistically significant. In contrast, the frequency of polyspecific TCR sequence usage was lower in female CD4 Tregs SP cells. These observations, which could be indicative of improved anti-infectious responses and worse autoimmune responses in women, warrant further exploration.

These inferences rely on a harmonized specificity compendium combining McPAS-TCR, IEDB, and VDJdb, which provide complementary and only partially overlapping landscapes (pathology-enriched versus predominantly viral specificities). Although there is limited numerical overlap between our thymic repertoires and annotated sequences, this is to be expected given that public databases currently capture only a small fraction of the potential TCR specificity space. In this context, the sex-biased enrichment of sequences annotated as autoimmune- and bacteria-associated should be viewed as a robust signal emerging from a very sparse annotation space. However, it should still be interpreted as hypothesis-generating rather than exhaustive.

To further evaluate the robustness of our findings, we performed additional analyses to assess whether donor age could confound the observed sex-specific differences. A potential limitation of our study is the relatively wide age range of donors. However, the absence of distinct age-related clustering in repertoire features, the lack of age impact on CD8 and CD4 Treg bacterial-specific TRB usage, and CD8 and CD4 Treg self-associated to AID-specific TRB usage patterns (Figure 8—figure supplement 3) argue against age as a confounding factor. These results support the hypothesis of an intrinsic sex bias in thymic TCR selection rather than an age-related effect.

While our findings provide valuable insights into sex-specific differences in thymic selection of TCR repertoires, our TCR specificity analyses were limited. We can confirm that our study focused exclusively on the TRB CDR3aa region of the TCR, which is widely acknowledged as the most critical element in defining TCR specificity (Springer et al., 2021; Korpela et al., 2023), while both the TRA and the TRB chains contribute to TCR specificity (Springer et al., 2021; Korpela et al., 2023). This limitation is intrinsic to the limited number of TRA with assigned specificity in databases and to the fact that, given the nature of our studies, there is a need to study large repertoires, which is currently not feasible by single-cell TCR sequencing. However, as methods for inferring specificity continue to evolve, they will likely allow more refined analyses of our dataset in the future (Ghoreyshi and George, 2023). Furthermore, the continuous development of the TCR database should enhance these analyses.

Despite the relatively small size of our cohort, which was outbred, we believe that this design is essential for capturing sex-associated differences. Inbred models would reduce inter-individual variability, but they would not reflect the complexity of human thymic selection, nor the interplay between sex and genetic diversity. Despite the modest sample size, the consistency of the observed patterns across the analytical approaches supports the robustness of our conclusions.

Altogether, our analyses of TCR specificity identified a biased selection in females towards sequences associated with AIDs, supporting the idea that early central tolerance mechanisms may represent one layer contributing to sex differences in autoimmune susceptibility, in interaction with peripheral and environmental factors. Future research should focus on validating these observations in relevant independent datasets, which are currently lacking.

## Methods

### Samples

Human thymus samples were obtained from 22 donors aged between 73 days and 64 years, without any specific pathologies (Figure 1). Individual partial HLA typing is detailed in Supplementary file 2. Pediatric thymus samples were obtained from children undergoing clinically indicated cardiac surgery at Necker Hospital, Paris. Adult samples were collected post-surgery at the Cardiac Surgery Departments of several hospitals in France from deceased donors, through an authorized tissue donation procedure approved by the Agence de la biomédecine. The collection and scientific use of human thymus samples were conducted in accordance with French regulations and under applicable authorization from the Agence de la Biomédecine and the French Ministry of Research (approval number #PFS14-009). All samples were de-identified prior to analysis. The ratio of male to female across donors is maintained at 1:1, although it varies between 1.1:1 and 1.33:1 depending on the cell subtypes analyzed. The age distributions of male and female donors were compared using two-sample Kolmogorov–Smirnov tests, with no significant differences observed across thymic subsets (*P* > 0.46), indicating no age-related confounding (Figure 1—figure supplement 1).

### Thymocyte isolation and RNA extraction

Thymic cell suspensions were prepared through automated tissue dissociation using gentleMACS dissociators (Miltenyi) or by mechanical disruption, followed by filtration through a 70 µm nylon mesh. The cells were stained with anti-CD3 Alexa Fluor 700 (BD Pharmingen, #561027), anti-CD4 APC (BD Pharmingen, #564976), anti-CD8 FITC (BD Pharmingen, #561948), and for some samples, anti-CD25 PE (BD, #341011) antibodies. Cell sorting was performed by fluorescent activated cell sorting (FACS) on a Becton Dickinson FACSAriaII, achieving a purity of >85%. The sorted populations included DP CD3+ (CD3 +CD4+CD8+), SP CD8+ (CD3 +CD4 CD8+), and SP CD4+ (CD3 +CD4+CD8-). A further separation was made into SP CD4 Teff (CD3 +CD4+CD8-CD25-) and SP CD4 Treg (CD3 +CD4+CD8 CD25+) for sixteen of the samples (Figure 1). For the sorted SP CD4, the Treg percentage varied between 5.8% and 16% across samples, with an average of 9.63%. Six samples of SP CD4 samples were not sorted into Teffs and Tregs; we thus categorized them as SP CD4 Teffs, assuming a purity of Teffs within the total SP CD4 of around 90%. Please refer to the detailed sample metadata in Supplementary file 3 of the supplementary material. This includes donor IDs and the number of sorted cells per thymic subset for each donor.

The RNA was isolated using the RNAqueous extraction kit (Invitrogen), in accordance with the manufacturer’s protocol. The RNA samples were quantified, and the integrity of each sample was determined using a Nanodrop (Thermo Fisher) or a Tapestation 4200 (Agilent, RNA ScreenTape).

### TCR library preparation and sequencing

TCR library preparation and sequencing were performed as described by Barennes et al., 2021. Briefly, RNA was processed using the SMARTer Human TCR a/b profiling v1 kit (Takarabio), in accordance with the manufacturer’s protocol. Amplicons were then purified using AMPure XP beads (Beckman Coulter), and their quantity was determined. The integrity of the amplicons was then assessed using the 2100 Bioanalyzer System (Agilent, DNA 1000 kit) or TapeStation 4200 (Agilent, D1000 screentape). Bulk Next-Generation Sequencing was performed using either a HiSeq 2500 (Illumina) with the SR-300 protocol plus 10% PhiX on the LIGAN-PM Genomics platform (Lille, France), or a NovaSeq 6000 (Illumina) with the PE-250 protocol plus 10% PhiX on the LIGAN-PM Genomics platform, or ICM platform (Paris, France).

### Data processing

The raw FASTQ/FASTA files were aligned to TRA and TRB using MiXCR (v3.0.13, RNA-seq parameters), a software solution that is able to correct for both sequencing and PCR errors (Bolotin et al., 2015). Samples with fewer than 1,000 CDR3aa sequences, an imbalanced TRA/TRB ratio (<1/9), or a low sequence count relative to sorted cell counts (<0.9 ratio) were removed. Sequences with CDR3aa lengths outside 6–23 amino acids were also discarded. To standardize clonotype counts and the number of unique clonotypes across sequencing techniques, the initial counts for each clonotype were reduced by 1 to exclude those with counts of 0 (singletons).

Furthermore, due to the high similarity between certain TRBV sequences, which has the potential to influence the analysis depending on the sequencing method used, TRBV06-2 and TRBV06-3 were merged as TRBV06-2/3, and TRBV12-3 and TRBV12-4 were combined as TRBV12-3/4 (Omer et al., 2022). Please refer to the summary table provided in the supplementary material (Supplementary file 3) for a comprehensive overview of the total number of TCR sequences retained after preprocessing, as well as the corresponding number of unique clonotypes, for each donor, chain, and thymic subset.

### Gene usage analysis

For both TRA and TRB chains across all cell subtypes, we analyzed the V and J gene usage and those of the VJ gene associations (Figure 1).

The usage of gene is calculated as follows:

$$\displaystyle \frac{\Sigma i\epsilon G^{ci}}{\Sigma j\epsilon S^{cj}}$$

Where:

- $\Sigma i\epsilon G^{ci}$ represents the total number of counts for clonotypes associated with the specified gene or gene association.
- $\Sigma j\epsilon S^{cj}$ represents the total sum of the counts of all clonotypes in the sample.

In this notation, G is the set of clonotypes using the interested gene, S is the set of all clonotypes in the sample, and c*_i_* and c*_j_* are the counts of the clonotypes *i* and *j*, respectively.

The usage of the V gene and the VJ gene combinations was investigated through dimensional reduction using PCA (MASS package v7.3–60, factoMineR package v2.8, factoextra package v1.0.7). Confidence ellipses were represented with a 5% alpha risk. Furthermore, for VJ gene combinations usage, a heatmap was constructed using the JSD distance to measure the distance between samples based on their gene usage distribution (complexHeatmap package v2.16.0, philentropy package v0.7.0). Hierarchical clustering was performed using the Euclidean distance and the complete linkage method.

### Diversity analysis

For the TCR repertoire diversity analysis, to correct for sequencing depth bias and differences in cellular richness between individuals, rarefactions were performed fifty times for each sample, using X clonotypes, where X represents the effective diversity number (Chaara et al., 2018).

The effective diversity was calculated as the exponential of the Shannon index ($e^{H}$) (Shannon, 1997).

Rényi indices (11 indices from 0 to Infinity) were calculated for each rarefaction (Rényi, 1961).

The median Shannon index, Simpson index, and Berger-Parker indices were then compared between the sexes.

### Distribution of CDR3aa length analysis

The calculation of the usage of all CDR3 by the amino acid length is as follows:

$$\displaystyle \frac{\Sigma i\epsilon L^{ci}}{\Sigma j\epsilon S^{cj}}$$

Where

- $\Sigma i\epsilon L^{ci}$ represents the sum of the counts of the CDR3 characterized by the amino acid length of interest.
- $\Sigma j\epsilon S^{cj}$ represents the total sum of counts of all CDR3s per sample.

In this notation, L is the set of CDR3s characterized by the amino acid length of interest, S is the set of all CDR3s in the sample, and c*_i_* and c*_j_* are the counts of the CDR3s *i* and *j*, respectively.

### Composition usage of amino acid within the CDR3 region

All CDR3aa sequences were aligned using the IMGT convention (positions p104-p118) (Lefranc et al., 2003).

Three complementary scales were used in the study. Firstly, sequence-level amino acid composition. Within the CDR3 loop region (IMGT positions p108-p114), involved in MHC-peptide complex interaction (Lefranc et al., 2003), amino acid usage at the sequence level was quantified for CDR3aa sequences 9–21 aa long in both TRA and TRB chains. For each sequence, the percentage usage of a given amino acid was calculated as follows:

(1) CDR3aa loop usage (%) = $\frac{n_{aa}}{L} \times 100$

Where $n_{aa}$ is the number of occurrences of the amino acid of interest in the CDR3 loop, and *L* denotes the total length of the CDR3aa sequence. Furthermore, amino acids were categorized into three hydropathy classifications (neutral, hydrophobic, and hydrophilic) using the IMGT classification that takes into account the Kyte-Doolittle hydropathy index (Pommié et al., 2004; Kyte and Doolittle, 1982)

Secondly, the position-specific amino acid usage at p109 and p110. To assess positional composition, for each individual, CDR3aa usage is examined at IMGT positions p109 and p110, corresponding to p6-p7 positions described by Stadinsky et al. and Khosravi-Maharlooei et al., which hydrophobic amino acids at these two central positions have been implicated in modulating TCR self-reactivity and cross-reactivity (Khosravi-Maharlooei et al., 2019; Lu et al., 2019; Stadinski et al., 2016). For a hydrophobic amino acid h (excluding alanine, due to its weak hydrophobicity) at position p, usage was defined:

(2) Hydrophobic CDR3aa usage in position (%) = $\frac{N_{\left (h,p\right)}}{n}\times 100$

Where n is the total number of unique CDR3 sequences and $N_{\left (h,p\right)}$ the number of unique CDR3 sequences carrying hydrophobic amino acid a at position p. This analysis quantifies amino acid composition at fixed positions independently of clonotype frequency.

### Probability of generation analysis

The probability of generation analysis of DP and CD8 cell subtypes consisted initially in the creation of a generative model of V(D)J recombination using 100,000 randomly selected non-productive sequences from all samples for both TRA and TRB chains, with the IGOR tool (Marcou et al., 2018). The generation probabilities (Pgen) for all sequences in the dataset were subsequently calculated using the OLGA tool, based on the respective generative models for each cell subtype and TCR chain (Sethna et al., 2019). Comparisons of Pgen values at the nucleotide level between males and females were performed. To ensure statistical robustness, control groups with equivalent male and female sample numbers were created by permuting the samples into two groups 20,000 times.

### TCR network structure by sequence similarity

For each sample, 100 random sub-samplings of CDR3aa sequences were performed, using the minimum CDR3aa count per cell subtype (refer to Supplementary file 1). Two CDR3aa were connected if their Levenshtein distance was = 1 (Figure 1; Madi et al., 2017). Network analysis focused on two metrics: the proportion of connected sequences and network density, defined as the ratio of actual connections to all possible connections. Median values from 100 subsampling iterations were then analyzed. Levenshtein distances were computed with the stringdist package (v0.9.10), networks were visualized using Cytoscape (v3.8.2), and network density was calculated with the igraph package (v1.5.1).

### TRB CDR3aa motif search

Two types of structural motifs were searched: local motifs (strict sequential motifs) and global motifs (sequences with substitutions preserving a positive BLOSUM62 score) (Figure 1).

The selection of enriched motifs was carried out using the gliph2 function with the turboGliph package (v0.99.2), with specific parameters defined as follows:

- The input sequences were from the sex group of interest (male or female), with reference sequences matched for TRBV gene usage.
- It is imperative that CDR3aa sequences are longer than eight aa.
- Local motif lengths were restricted to 3–5 aa.
- Only motifs consisting of more than two unique CDR3aa were conserved.
- Local motif significance of Fisher’s test has not been boosted.

Additional filters were applied so that: (i) the inclusion of public CDR3aa sequences is a prerequisite for a motif, with these sequences shared by at least two individuals, (ii) a significant enrichment is required, as determined by Fisher’s test with a p-value <0.01 and (iii) a usage difference between groups of at least twofold is necessary, as evidenced by a Wilcoxon test with a p-value <0.05.

Motif validation was performed on an openly published pediatric thymic dataset from the Arstila team (Heikkilä et al., 2021; Mattila et al., 2023) consisting of a bulk TCR dataset from infants aged between 7 days and 8 months. Here, we removed one male subject, ‘Thymus A’, who was a twin of the ‘Thymus B’ and ‘Thymus 1’ neonate sample, in order to analyze height samples with a male-to-female ratio of 5:3. Further validation was conducted on sorted peripheral blood TCR repertoires (CD8, CD4 Teff, and CD4 Treg) from 75 healthy volunteers aged from 18 to 84 years (male-to-female ratio of 1.03:1), whose libraries were generated under the same conditions as those of our thymic dataset. They were recruited within the Transimmunom observational trial (NCT02466217) and HEALTHIL-2 (NCT03837093; Lorenzon et al., 2018). PBMCs from healthy individuals were isolated using Ficoll density gradient centrifugation and enriched for CD4+T cells via EasySep Human CD4+T Cell magnetic beads (Stemcell). The samples were then separated into two groups: effector T cells (CD4+CD25-) and Tregs (CD4+CD25+). This was done using EasySep Human Pan-CD25 magnetic beads (Stemcell). Purity was assessed by flow cytometry based on the expression of CD4 and FoxP3, with a purity threshold of >80%. From the frozen PBMC aliquots, CD8+T cells (CD3+CD8+) were sorted using a FACS ARIA II cell sorter.

### TRB CDR3aa specificity analysis

We have established a curated and harmonized TCR specificity database by integrating data from three public TCR repositories: Mc-PAS, IEDB, and VDJdb (Tickotsky et al., 2017; Shugay et al., 2018; Vita et al., 2019; data collected as of October 2023, and accessible at https://doi.org/10.5281/zenodo.20402420; Figure 1). The harmonization procedure was carried out in accordance with the framework outlined in Jouannet et al., 2025 and included:

- Uniform formatting of TRA/TRB TCR sequences and associated metadata,
- Consolidation of redundant entries (identical TRA/TRB TCR, epitope, organism, PubMed ID, and cell subset),
- Maintaining separate entries when source databases are incompatible,

Reliability levels for sequences and specificity assignments were quantified using the Verified_score (VS) and Antigen-identification_score (AIS) as defined in Jouannet et al., 2025. The VS, ranging from 0 to 2, reflects the concordance between calculated and curated CDR3 boundaries following the IEDB verification strategy (VS = 2: both TRA and TRB present and verified; VS=1.1: only TRA verified; VS=1.2: only TRB verified; VS=0: no verified chain) and the AIS (0–5), which ranks the strength of antigen-identification methods (Jouannet et al., 2025).

In this study, we focused our analyses on high-reliability TRB sequences, defined as those with a verified TRB CDR3aa (VS ≥1.2) and an AIS corresponding to in vitro T cell stimulation with a pathogen, protein, or peptide, or pMHC X-mer sorting (AIS >3.2, excluding categories 4.1 and 4.2). This ensures that the specificity assignments relied on robust experimental evidence.

Specificity groups were categorized according to antigen class, encompassing viral, bacterial, yeast, parasitic, and self-antigens along with antigens derived from wheat, plants, or animals. Self-antigens were then annotated into three categories: autoimmune-associated antigens, cancer-associated antigens, and self-antigens unrelated to disease. For autoimmune-associated antigens, assignments were based on explicit annotations present in McPAS-TCR, IEDB, and VDJdb. These databases identify celiac disease (CeD) and type 1 diabetes (T1D) related antigens, which were retained as such in the harmonized dataset. For cancer-associated antigens, the following classifications were used: (i) information present in the original databases and (ii) cross-referencing with external cancer antigen resources, including the Cancer Antigenic Peptide Database (de Duve Institute), the Human Protein Atlas, and the cancer database caAtlas (Yi et al., 2021). A comprehensive list of all self-antigens included in the filtered harmonized database, together with their associated disease category and their mapping into the specificity groups used, is provided in Supplementary file 4.

This harmonized database was used to identify exact matches with the CDR3aa sequences of our TRB sequences. The enrichment of CDR3aa sequences associated with a given specificity group in male and female thymic repertoires for each cell subtype compared to the specificity distribution in the database was tested. Furthermore, we performed specificity group distributions between male and female samples, as well as the overall usage of these sequences across entire TCR repertoires.

Additionally, our research concentrated on polyspecific sequences defined as CDR3aa TRB sequences recognizing at least two distinct microbial species or belonging to multiple specificity groups for non-microbial antigens. In this study, polyspecificity is therefore considered at the level of broader antigenic categories rather than individual epitopes and thus reflects functional cross-reactivity patterns aggregated across studies, instead of implying molecular-level multi-epitope recognition. These polyspecific TRB CDR3aa sequences underwent the same statistical analyses as monofunctional sequences.

### Statistical analysis

For VJ gene usage, CDR3aa length usage, CDR3aa usage, network similarity, and specificity analyses, the comparison between males and females was statistically analyzed using the Wilcoxon test. The statistical tests were performed using the ggpubr library (v0.6.0) in R. The number of asterisks denotes the significance of the results: one asterisk indicates a p-value ≤0.05, two asterisks indicate a p-value ≤0.01, and so on until four asterisks, denoting a p-value ≤0.0001. The comparisons of the Rényi curves and Pgen were compared using the Kolmogorov-Smirnov test. The Wilcoxon test was used to test the comparisons of the proportions of specific sequences in male and female subjects relative to those in the specificity database.

## Data Availability

All raw sequencing data generated in this study are publicly available on NCBI under the BioProject accession PRJNA1379632 (https://www.ncbi.nlm.nih.gov/bioproject/PRJNA1379632/). The following dataset was generated: KlatzmannD Mariotti-FerrandizE 2025TRiPoD_human_TCRNCBI BioProjectPRJNA1379632 The following previously published dataset was used: HeikkilaN KleinoI VanhanenR YohannesDA MattilaIP SaramakiJ ArstilaP 2021Characterization of human T cell receptor repertoire data in eight thymus samples and four related blood samplesENA European Nucleotide ArchivePRJEB4193610.1016/j.dib.2021.106751PMC785929233553521

## Additional files

Supplementary file 1. Minimal number of CDR3aa by cell subset.

Supplementary file 2. HLA typing of the study cohort. HLA class I (A, B, C) and class II (DP, DQA, DQB, DR) alleles for each subject included in the analysis. Due to the clinical nature of the data, typing is provided at low resolution (first-field/serological equivalent). "Not collected" indicates loci that were not documented in the donor’s clinical file at the time of organ procurement.

Supplementary file 3. Detailed sample-level overview of the thymic cohort. For each donor and thymocyte subset, the table reports: donor ID, sex, age at sampling, thymocyte subset (DP, CD8 SP, CD4 Teff SP, CD4 Treg SP), number of cells sorted, and for each TCR chain (TRA and TRB), the total number of CDR3 amino acid sequences retained after preprocessing (quality filtering, length filtering, and MiXCR-based error correction) and the corresponding number of unique clonotypes. This table provides the metadata referred to in the Samples, Thymocyte isolation and RNA extraction, and Data processing sections, and complements the global cohort description shown in Figure 1.

Supplementary file 4. Annotation of self-antigens and their associated disease contexts. This table lists the self-antigens included in the curated TCR specificity database, together with their associated diseases, when applicable, and their assignment to the corresponding specificity class used in the analyses. MAA = Melanoma associated antigen, TAA = Tumor associated antigen, DT1=type 1 diabetes, CeD = celiac disease.

MDAR checklist
