# Supplementary material for: Thymic selection of the T cell receptor repertoire is biased toward autoimmunity in females
Source: eLife. 2026 Aug 20;14:RP109041. doi: 10.7554/eLife.109041 (PMC13493126; doi:10.7554/eLife.109041)
Supplement: Peer review history

# Editor report: eLife Assessment

**DOI:** 10.7554/eLife.109041.4.sa0

**Authors:** Juan Carlos Zúñiga-Pflücker

**Affiliations:** University of Toronto Canada

**Keywords:** Incomplete, Valuable

This study provides **valuable** insights into addressing the question of whether the prevalence of autoimmune disease could be driven by sex differences in the T cell receptor (TCR) repertoire, correlating with higher rates of autoimmune disease in females. The authors compared male and female TCR repertoires using bulk RNA sequencing, from sorted thymocyte subpopulations in pediatric and adult human thymuses; however, the analyses provided do not provide sufficient discrimination, as paired TCR chains are not examined, and **incompletely** support the central claims regarding sex differences in the TCR repertoire and potential autoimmune bias.

# Referee report: Reviewer #2 (Public review)

**DOI:** 10.7554/eLife.109041.4.sa1

Summary

This study addresses the hypothesis that the higher prevalence of autoimmune diseases in women could result from sex-dependent differences in thymic generation or selection of TCR repertoires. The biological question is important and the dataset is valuable. However, the study has major conceptual and analytical limitations.

In particular:

- The conclusions cannot be generalized to autoimmune diseases as a whole, as only type 1 diabetes (T1D) and celiac disease (CeD) antigens were analyzed.

- The central interpretation is not supported by the data, as the observed signal is strongly influenced by TCRs associated with T1D, which shows a male-biased incidence and therefore does not align with the female bias the study aims to explain.

Strengths

The key strength of this work is the newly generated dataset of TCR repertoires from sorted thymocyte subsets (DP and SP populations). This approach enables the authors to distinguish between biases in TCR generation (DP) and thymic selection (SP). Bulk TCR sequencing allows deeper repertoire coverage than single-cell approaches, which is valuable here. However, the absence of TRA-TRB pairing and HLA context limits the interpretability of antigen specificity analyses.

Weaknesses

The authors did not adequately address the central concerns raised in the previous review. As a result, the major issues remain unresolved.

(1) Generalization to autoimmune diseases is not justified.

The study aims to explain the higher prevalence of autoimmune diseases in females. The main conclusion is based on enrichment in females of TCRs annotated as autoimmune-associated using database matching.

However, these matches correspond exclusively to TCRs specific for T1D and CeD. This already limits the conclusions to these two diseases and does not justify generalization to autoimmune diseases as a whole.

(2) Contradiction with epidemiology of T1D which is male-biased

T1D and CeD have opposite sex biases in European populations. While CeD is more frequent in females (~60%; doi:10.1016/j.cgh.2018.11.013), T1D is more frequent in males (male:female = 1.11 in France; doi:10.1111/dom.70124).

Importantly, T1D constitutes a substantial fraction of the autoimmune-associated dataset (42 out of 48 epitopes; 83 out of 185 TRB sequences). Therefore, the observed signal is strongly influenced by a disease that does not follow the female bias the study aims to explain.

The authors argue that T1D sex bias varies globally, including female-biased incidence in East Asia and Africa. However, this argument does not resolve the issue, as the cohort analyzed in this study was derived from France, where T1D shows a male-biased incidence. Thus, the interpretation remains inconsistent with the population context of the dataset.

(3) Lack of disease-level and donor-level resolution

The authors combine T1D and CeD into a single "autoimmune" category and do not provide per-disease, per-donor or per-epitope distributions, despite explicit reviewer's requests.

This prevents evaluation of whether the observed signal is driven by:

- a specific disease (T1D or CeD), or

- a small number of donors

Without this analysis, the conclusions cannot be properly interpreted.

(4) Use of "polyspecificity" concept is not supported by experimental evidence

The authors extensively use the concept of "polyspecific TCRs," defined as single-chain CDR3 sequences annotated across databases as recognizing distinct and unrelated antigenic categories. This concept is not supported by experimental evidence (except for a single TCR in Quiniou et al., as acknowledged by the authors).

In the absence of robust validation, a more parsimonious explanation for such ambiguously annotated TCR chains is the presence of false-positive annotations in public databases (see, e.g., Ton Schumacher's preprint https://www.biorxiv.org/content/10.1101/2025.04.28.651095.abstract) or alternatively, distinct TRA pairing for identical TRB sequences resulting in different specificities.

The observation that these TCRs have high generation probability is expected, as TCRs found in independent studies are likely to have high generation probability. The interpretation of these sequences as biologically meaningful entities (e.g., a "first line of defense") is therefore speculative and not supported by the data.

The authors also refer to in silico-generated polyspecific TCRs (ref. to Nature Machine Intelligence). However, such sequences are generated ex vivo and do not undergo thymic selection. A TCR capable of recognizing multiple unrelated foreign antigens would likely also recognize self-antigens and be eliminated during negative selection. Therefore, this argument does not support the biological relevance and in vivo existence of the proposed polyspecific TCR class.

(5) Insufficient statistical analysis of diversity

The absence of statistically significant differences in repertoire diversity between sexes (Figure 3), despite an apparent visual trend, may reflect limited sample size and insufficient statistical power rather than a true absence of differences. A more appropriate statistical approach, such as mixed-effects modeling, was requested in the previous review but was not performed.

# Author comment: Author response

**DOI:** 10.7554/eLife.109041.4.sa2

**Authors:** Hélène Vantomme, Valentin Quiniou, Leslie Adda, Charline Jouannet, Vanessa Mhanna, Celine Albalaa, Pierre Barennes, Nicolas Coatnoan, Vimala Diderot, Johanna Dubois, Gwladys Fourcade, Kenz Le Gouge, Otriv Nguekap Tchoumba, Martin Pezous, Paul Stys, Adrien Six, Encarnita Mariotti-Ferrandiz, David Klatzmann

**Affiliations:** Sorbonne Université Paris France; Sorbonne Université, AP-HP, Hôpital Pitié-Salpêtrière Paris France; Sorbonne Université Paris France; Inserm Montgeron France; Sorbonne Université Paris France; Sorbonne Université Paris France; Sorbonne Université Paris France; AP-HP, Hôpital Pitié-Salpêtrière Paris France; Sorbonne Université Paris France; Sorbonne Université Paris France; Sorbonne Université Paris France; Sorbonne Université Paris France; Sorbonne Université Paris France; Sorbonne Université Paris France; Sorbonne Université Paris France; Sorbonne Université Paris France; Sorbonne Université Paris France; Sorbonne Université Paris France

The following is the authors’ response to the previous reviews

**Public Reviews:**

**Reviewer #1 (Public review):**

Summary:

The goal of this paper was to determine whether the T cell receptor (TCR) repertoire differs between a male or female human. To address this, this group sequenced TCRs from doublepositive and single-positive thymocytes in male and female humans of various ages. Such an analysis on sorted thymocyte subsets has not been performed in the past. The only comparable dataset is a pediatric thymocyte dataset where total thymocytes were sorted.

They report on participant ages and sexes, but not on ethnicity, race, nor provide information about HLA typing of individuals. The experiments are heroic, yet do represent a relatively small sampling of diverse humans. They observed no differences in TCRbeta or TCRalpha usage, combinational diversity, or differences in the length of the CDR3 region, or amino acid usage in the CD3aa region between males or females. Though they observed some TCRbeta CD3aa sequence motifs that differed between males and females, these findings could not be replicated using an external dataset and therefore were not generalizable to the human population.

They also compared TCRbeta sequences against those identified in the past databases using computational approaches to recognize cancer-, bacterial-, viral-, or autoimmune-antigens. They found little overlap of their sequences with these annotated sequences (depending on the individual, ranged from 0.82-3.58% of sequences). Within the sequences that were in overlap, they found that certain sequences against autoimmune or bacterial antigens were significantly over-represented in female versus male CD8 SP cells. Since no other comparable dataset is available, they could not conclude whether this is a generalizable finding in the human population.

Strengths:

It is a novel dataset that attempts to understand sex differences in the T cell repertoire in humans. Overall, the methodologies are sound and are the current state-of-the-art. There was an attempt to replicate their findings in cases where an appropriate dataset was available. I agree that there are no gross differences in TCR diversity between males and females. This is an important negative result.

Weaknesses:

Weaknesses:

Overall, the sample size is small given that it is an outbred population. This reviewer recognizes the difficulty in obtaining samples for this experiment (which were from deceased donors), and this limitation was appropriately discussed. Their analysis was limited by the current availability of other TCR sequences. These weaknesses were appropriately discussed and considered.

We thank this reviewer for his appreciation of our work.

**Reviewer #2 (Public review):**

Summary:

This study addresses the hypothesis that the strikingly higher prevalence of autoimmune diseases in women could be the result of biased thymic generation or selection of TCR repertoires. The biological question is important and the hypothesis is valuable. Although the topic is conceptually interesting and the dataset is rich, the study has a number of major issues. In particular, the majority of "autoimmunity-related TCRs" considered in this study are in fact specific to type 1 diabetes (T1D). Notably, T1D incidence is higher in males, which directly contradicts the stated objective of the study - to explain the higher prevalence of autoimmune diseases in women. Given this conceptual inconsistency, the evidence presented does not support the authors' conclusions.

We disagree with the reviewer’s assertion that our findings create a conceptual inconsistency.

Autoimmune diseases are multifactorial conditions in which multiple biological layers, including thymic selection, peripheral immune regulation, hormonal effects, environmental exposures, and tissue-specific vulnerability, contribute to disease incidence. These layers may influence sex ratios in different directions. Therefore, observing a higher frequency of TCRs annotated as T1D-associated in females does not imply that T1D incidence must also be higher in females.

Actually, T1D incidence itself is not uniformly male-biased worldwide. Epidemiological analyses (reviewed in Qu and Hakonarson, Diabetes Obes Metab 2025) show that male predominance is mainly observed in high-incidence Northern European populations, whereas in several lowerincidence regions, including parts of East Asia and Africa, the sex ratio is balanced or even femalebiased. Furthermore, another recent study highlights that T1D incidence and prevalence in women and men varies depending on the study period (PMC12544016).

This heterogeneity indicates that disease incidence reflects context-dependent interactions between genetic load, environmental exposures, and sex-specific biological modifiers. Moreover, biological sex acts as a dynamic modifier of genetic risk and immune function in T1D, influencing central tolerance, peripheral immune activation, and β-cell intrinsic resilience (reviewed in Qu and Hakonarson, 2025). Experimental models further demonstrate estrogenmediated protection of pancreatic β-cells (Kim et al., Biochem Biophys Res Commun 2025), indicating that disease incidence reflects the integration of immune, hormonal, and tissuespecific layers rather than central autoreactive TCR release alone. Sex hormones may exert distinct and sometimes opposing effects on thymic selection and on target-organ vulnerability, while environmental factors such as vitamin D status, infections, and microbiota composition further shape disease expression.

Importantly, our study does not claim causality, nor does it aim to predict the epidemiology of any specific autoimmune disease. Our conclusions are limited to the observation that sexdependent differences exist in thymic TCR selection.

Strengths:

The key strength of this work is the newly generated dataset of TCR repertoires from sorted thymocyte subsets (DP and SP populations). This approach enables the authors to distinguish between biases in TCR generation (DP) and thymic selection (SP). Bulk TCR sequencing allows deeper repertoire coverage than single-cell approaches, which is valuable here, although the absence of TRA-TRB pairing and HLA context limits the interpretability of antigen specificity analyses. Importantly, this dataset represents a valuable community resource and should be openly deposited rather than being "available upon request."

We agree with the reviewer’s comment. As already stated in the previous revision and the "Data Availability" section of the manuscript, all raw sequencing data have been deposited and are publicly available on NCBI (BioProject PRJNA1379632): https://www.ncbi.nlm.nih.gov/sra/PRJNA1379632.

Weaknesses:

I thank the authors for their detailed responses to my previous comments. Several concerns were addressed satisfactorily; however, important issues remain unresolved, and a new major concern has emerged from the revised manuscript.

Major concerns:

(1) Autoimmune specificity is dominated by T1D, contradicting the study's premise. Newly added supplementary Table 3 shows that the authors considered only 14 autoimmune-related epitopes, of which 12 are associated with type 1 diabetes (T1D) and 2 with celiac disease (CeD). (I guess this is because identification of particular peptide autoantigens is an extremely difficult task and was only successful in T1D and CeD.) Thus conclusions of this work mostly relate to T1D. However, the incidence of T1D is higher in males than in females (e.g. doi:10.1111/j.13652796.2007.01896.x; doi:10.25646/11439.2). This directly contradicts the stated objective of the study - to explain the higher prevalence of autoimmune diseases in women. As a result, the authors' conclusions (a) cannot be generalized to autoimmune disease as a whole as the authors only considered T1D and CeD antigens and (b) are internally inconsistent with the stated objective of the study.

(2) By contrast, CeD does show a female bias (~60/40 female/male; doi: 10.1016/j.cgh.2018.11.013). However, the manuscript does not allow evaluation of how much the reported "autoimmune TCR enrichment" derives from T1D versus CeD. Despite my previous request, the authors did not provide per-donor and per-epitope distributions of autoimmune-specific TCR matches. I therefore explicitly request a table in which: each row corresponds to a specific autoimmune antigen; each column corresponds to a donor (with metadata available including sex); each cell reports the number of unique TCRs specific to that antigen in that donor. Without such data, the conclusions cannot be evaluated.

(3) It is scientifically inappropriate to generalize findings to "autoimmune diseases" when only T1D and CeD were analyzed. Moreover, given that T1D and CeD show opposite directions of sex bias, combining them into a single "AID" category is misleading. All analyses presented in Figure 8 and Supplementary Figure 16 should be repeated and shown separately for T1D and CeD, rather than combined.

We acknowledge that currently available antigen-annotated TCR databases remain limited. This reflects the considerable experimental difficulty of defining TCRs’ antigen specificities and is a widely recognized limitation in the field.

In the curated database used here, the autoimmune-associated entries correspond primarily to type 1 diabetes (T1D) and celiac disease (CeD), two autoimmune contexts for which antigen-specific TCRs have been experimentally characterized. However, focusing on the number of antigens alone does not accurately reflect the breadth of the dataset.

Specifically, our analysis is based on 48 epitopes and nearly 200 annotated TRB sequences, providing substantially broader antigenic representation than suggested by antigen count alone.

**Author response table 1.** 

Disease	Antigens	Epitopes	TRB CDR3 sequences
Type 1 diabetes	12	42	83
Celiac disease	2	6	102

Importantly, our analytical framework does not attempt to interpret each epitope specificity individually. Instead, we examine whether TCRs annotated as autoimmune-associated are differentially represented between sexes at the level of thymic selection.

In our dataset we observe a stronger CD8⁺ thymic selection of TCRs annotated as autoimmune- associated in females. We interpret this as evidence that central tolerance mechanisms may contribute to sex-dependent differences in autoreactive repertoire composition, rather than as a determinant of any specific autoimmune disease pathophysiology.

(4) The McPAS database contains TCRs associated with other autoimmune diseases (e.g., multiple sclerosis, rheumatoid arthritis), although the exact autoantigens in these contexts are unknown. Why didn't the authors perform the search for such TCRs? I believe disease association even without particular known antigen could still be insightful.

For multiple sclerosis, the only antigen present in the database is myelin basic protein (MBP). In our thymic repertoire dataset, we could not detect any CDR3 sequence matching MPB annotated CDR3s from the database.

For rheumatoid arthritis, the database contains only a small number of TRA sequences without corresponding TRB chains. Because our specificity analysis is based on TRBs, these entries could not be used in our analyses.

(5) Misuse of the concept of polyspecificity. I appreciate the authors' reference to Don Mason's work; however, the concept of polyspecificity discussed there is fundamentally different from the authors' usage. Mason, Sewell (doi:10.1074/jbc.M111.289488), Garcia(doi:10.1016/j.cell.2014.03.047), and others demonstrated that individual TCRs can recognize multiple peptides, possibly around 1 million. But importantly these peptides are not random but share some sequence motif. This is a general feature of TCRs, i.e. 100% of TCRs are polyspecific in this sense.

In contrast, the authors define polyspecificity as TRB sequences annotated as specific to unrelated epitopes in TCR databases such as VDJdb. These databases are well known to contain substantial numbers of false-positive annotations (see, e.g., Ton Schumacher's preprint https://www.biorxiv.org/content/10.1101/2025.04.28.651095.abstract). The authors acknowledge that, under their definition, polyspecificity has been experimentally validated for only one (!) TCR (Quiniou et al.). In the absence of robust experimental validation, use of the term "polyspecificity" in this context is misleading. I strongly recommend removing all analyses and conclusions related to polyspecificity from the manuscript unless supported by independent functional validation.

We agree with the reviewer that the concept of TCR polyspecificity is complex, controversial and not uniformly defined in the literature.

For some, polyspecificity refers to the ability of individual TCRs to recognize multiple related peptides sharing structural motifs, as described by Mason, Sewell, Garcia, and others. With this definition, we agree that many/most TCRs exhibit some degree of cross-reactivity and would thus be defined as polyspecific.

In contrast, our definition of polyspecificity came from our observation arising from large-scale repertoire analyses that certain CDR3 sequences are repeatedly annotated across databases as recognizing distinct and unrelated antigenic categories. In our previous study (Quiniou et al.), we showed that these sequences display specific biochemical and repertoire features and may represent a particular class of TCRs involved in early or heterologous immune responses. A classic cross reactivity based on structural motif sharing could not explain these results.

We believe that the existence of such TCRs, rather than classic cross-reactive TCRs, has the potential to better explain why patients with extremely reduced TCR repertoires (around 3000 TCRs only) can respond well to various infectious challenges (https://doi.org/10.1073/pnas.97.1.274) or why there are T cells with memory phenotypes against viruses not previously encountered (https://pmc.ncbi.nlm.nih.gov/articles/PMC3626102/). We acknowledge that direct experimental validation of the function of such TCRs is currently limited; further work will help clarify the notion of polyspecificity, and hopefully to better understand the overlooked “heterologous immunity”.

Of note, a recent paper in Nature Machine Intelligence (https://doi.org/10.1038/s42256-02501096-6) described the *in-silico* generation of antigen-specific TCRs. Using our definition of polyspecificity (TCRs with higher generation probabilities, specific V/J gene preferences, shared CDR3s across individuals, and reactivity to multiple unrelated peptides), they showed that “multitask models preferentially sample polyspecific CDR3β sequences”. Therefore, we consider the debate on polyspecificity to be ongoing, and our discussion of polyspecificity in this paper to be part of this debate.

(6) I agree that comparing specificity enrichment between sexes is meaningful. However, enrichment relative to the database composition itself is not biologically interpretable, as acknowledged by the authors in their response. I therefore recommend removing Supplementary Figure 15, which is potentially misleading.

In the original manuscript, the comparison to the pooled database was intended as a descriptive assessment rather than as a biological enrichment analysis. Differences between an experimental thymic repertoire and a curated reference database are expected, given the structure and annotation biases inherent to the reference resource.

The purpose of Supplementary Figures 15B and 15C was therefore twofold: (i) to provide a descriptive overview of how specificity categories are distributed in our thymic dataset relative to the curated database, and (ii) to evaluate whether deviations from database proportions were of similar magnitude in males and females, ensuring that database composition did not differentially bias one sex over the other. In addition, the donor-resolved representations demonstrate that these patterns are consistent across individuals and are not driven by a single donor.

To avoid any potential misinterpretation, we have revised the manuscript to remove references to “enrichment” relative to database composition and eliminated quantitative comparisons to baseline database frequencies. The corresponding text and figure legends have been clarified to indicate that these analyses are descriptive and methodological in nature, while all biological interpretations rely exclusively on direct sex-specific comparisons within the thymic dataset.

(7) In contrast, Supplementary Figure 16 represents the most convincing result of the study (keeping in mind that the AID group should be splitted to T1D and CeD with T1D and that T1D and CeD have opposing directions of sex biases) and should be shown as a main figure, replacing Figure 8A-B which is less convincing as it doesn't show per-donor distribution.

(8) The authors argue that applying mixed-effects modeling to Rényi entropy would require assuming a common sex effect across subsets. I do not find this assumption unreasonable. For example, if sex effects are mediated through AIRE-dependent negative selection, one would indeed expect a consistent direction of effect across subsets. The lack of statistical significance in Figure 3 may reflect limited sample size rather than true absence of the difference. Moreover, the title's phrasing "comparable TCR repertoire diversity" is vague: what is the statistical definition of "comparable"?

The use of “comparable” in comparing TCR repertoire diversity is indeed “soft”, and aimed to indicate that there are no obvious dissimilarities.

**Recommendations for the authors:**

**Reviewer #2 (Recommendations for the authors):**

Minor comments:

(1) Available HLA typing data for selected donors should be included as a table in the manuscript.

The available low-resolution HLA typing data for the donors included in this study have been compiled and added as Supplementary Table 1 in the revised manuscript.

(2) The authors' explanation for why external validation of gene usage biases was not possible should be concisely incorporated into the Discussion.

We have incorporated a concise explanation in the Discussion clarifying why independent validation of the TRBV6-5 bias in external thymic datasets is currently not feasible, due to the absence of publicly available cohorts combining sorted thymic subsets, balanced sex representation, and sufficient sequencing depth.

(3) The clarification that considered sex-specific motifs are public should be included explicitly in the main text, not only figure legend and methods.

We now explicitly state in the main Results section that only public motifs, defined as motifs containing CDR3 sequences shared by at least two individuals, were retained in the analysis.

(4) The statement "Thymocytes expressing TCRs with insufficient or excessive avidity are eliminated (negative selection)" is strictly speaking incorrect. Thymocytes with insufficient avidity are eliminated by death by neglect during positive selection.

We thank the reviewer for pointing out this imprecision. The statement has been corrected.

(5) Figure 8C is unclear - what does "80% of unique polyspecific TCRs" mean? In any case, I strongly recommend removal of all polyspecificity-related analyses.

We apologize for the lack of clarity in the axis label of Figure 8C. To clarify, this analysis represents the proportion of polyspecific CDR3aa sequences among all sequences with an assigned specificity within an individual’s repertoire. Specifically, it measures how many unique TCR sequences, previously identified as having a known specificity in reference databases, are also categorized as polyspecific.

To address the reviewer’s concern, we have updated the Y-axis label of Figure 8C to: "Proportion of polyspecific CDR3aa among antigen-specific sequences (%)".

(6) "However, no significant sex-based differences were found in the usage of hydrophobic, hydrophilic, or neutral aa at the critical p109 and p110 positions in TRB" - this Discussion statement is inconsistent with the new analysis on Fig. 4C.

We regret that the Discussion still contained wording from a previous version of the analysis. The text has now been corrected to reflect the updated results showing a significant increase in hydrophobic amino acid usage at positions p109/p110.

(7) In the Discussion the authors write: "the absence of age-related clustering in repertoire features (data not shown)". What is the reasoning for not showing the data?

We understand the reviewer's point. This exploratory clustering analysis was performed on the data presented in the heatmaps (Figure 2B and Supplemental Figures 10-13). However, as it revealed no distinct patterns or clustering based on the donors' age (with samples from different age groups being interspersed throughout the clusters), we chose not to add an extra layer of annotation to Figure 2B to maintain clarity.
